# A proteome-scale map of the SARS-CoV-2–human contactome

**DOI:** 10.1038/s41587-022-01475-z

**Published:** 2022-10-10

**Authors:** Dae-Kyum Kim, Benjamin Weller, Chung-Wen Lin, Dayag Sheykhkarimli, Jennifer J. Knapp, Guillaume Dugied, Andreas Zanzoni, Carles Pons, Marie J. Tofaute, Sibusiso B. Maseko, Kerstin Spirohn, Florent Laval, Luke Lambourne, Nishka Kishore, Ashyad Rayhan, Mayra Sauer, Veronika Young, Hridi Halder, Nora Marín-de la Rosa, Oxana Pogoutse, Alexandra Strobel, Patrick Schwehn, Roujia Li, Simin T. Rothballer, Melina Altmann, Patricia Cassonnet, Atina G. Coté, Lena Elorduy Vergara, Isaiah Hazelwood, Betty B. Liu, Maria Nguyen, Ramakrishnan Pandiarajan, Bushra Dohai, Patricia A. Rodriguez Coloma, Juline Poirson, Paolo Giuliana, Luc Willems, Mikko Taipale, Yves Jacob, Tong Hao, David E. Hill, Christine Brun, Jean-Claude Twizere, Daniel Krappmann, Matthias Heinig, Claudia Falter, Patrick Aloy, Caroline Demeret, Marc Vidal, Michael A. Calderwood, Frederick P. Roth, Pascal Falter-Braun

**Affiliations:** 1 Donnelly Centre for Cellular and Biomolecular Research (CCBR), University of Toronto, Toronto, Ontario Canada; 2 Department of Molecular Genetics, University of Toronto, Toronto, Ontario Canada; 3 Lunenfeld-Tanenbaum Research Institute (LTRI), Sinai Health System, Toronto, Ontario Canada; 4 Center for Cancer Systems Biology (CCSB), Dana-Farber Cancer Institute, Boston, MA USA; 5 Department of Cancer Genetics and Genomics, Roswell Park Comprehensive Cancer Center, Buffalo, NY USA; 6 Institute of Network Biology (INET), Molecular Targets and Therapeutics Center (MTTC), Helmholtz Zentrum München, German Research Center for Environmental Health, Munich-Neuherberg, Germany; 7 Unité de Génétique Moléculaire des Virus à ARN, Département de Virologie, Institut Pasteur, Paris, France; 8 UMR3569, Centre National de la Recherche Scientifique, Paris, France; 9 Université de Paris, Paris, France; 10 Aix-Marseille Université, Inserm, TAGC, Marseille, France; 11 Institute for Research in Biomedicine (IRB Barcelona), Barcelona Institute for Science and Technology, Barcelona, Spain; 12 Research Unit Cellular Signal Integration, Institute of Molecular Toxicology and Pharmacology, Molecular Targets and Therapeutics Center (MTTC), Helmholtz Zentrum München, German Research Center for Environmental Health, Munich-Neuherberg, Germany; 13 Laboratory of Viral Interactomes, GIGA Institute, University of Liège, Liège, Belgium; 14 Department of Genetics, Blavatnik Institute, Harvard Medical School, Boston, MA USA; 15 Department of Cancer Biology, Dana-Farber Cancer Institute, Boston, MA USA; 16 TERRA Teaching and Research Centre, University of Liège, Gembloux, Belgium; 17 Laboratory of Molecular and Cellular Epigenetics, GIGA Institute, University of Liège, Liège, Belgium; 18 Molecular Architecture of Life Program, Canadian Institute for Advanced Research (CIFAR), Toronto, ON Canada; 19 CNRS, Marseille, France; 20 Institute of Computational Biology (ICB), Computational Health Center, Helmholtz Zentrum München, German Research Center for Environmental Health, Munich-Neuherberg, Germany; 21 Department of Informatics, Technische Universität München, Munich, Germany; 22 Institució Catalana de Recerca I Estudis Avaçats (ICREA), Barcelona, Spain; 23 Department of Computer Science, University of Toronto, Toronto, Ontario Canada; 24 Microbe-Host Interactions, Faculty of Biology, Ludwig-Maximilians-Universität (LMU) München, Planegg-Martinsried, Germany

**Keywords:** Biochemical networks, Disease genetics, NF-kappaB, Viral infection, Target identification

## Abstract

Understanding the mechanisms of coronavirus disease 2019 (COVID-19) disease severity to efficiently design therapies for emerging virus variants remains an urgent challenge of the ongoing pandemic. Infection and immune reactions are mediated by direct contacts between viral molecules and the host proteome, and the vast majority of these virus–host contacts (the ‘contactome’) have not been identified. Here, we present a systematic contactome map of severe acute respiratory syndrome coronavirus 2 (SARS-CoV-2) with the human host encompassing more than 200 binary virus–host and intraviral protein–protein interactions. We find that host proteins genetically associated with comorbidities of severe illness and long COVID are enriched in SARS-CoV-2 targeted network communities. Evaluating contactome-derived hypotheses, we demonstrate that viral NSP14 activates nuclear factor κB (NF-κB)-dependent transcription, even in the presence of cytokine signaling. Moreover, for several tested host proteins, genetic knock-down substantially reduces viral replication. Additionally, we show for USP25 that this effect is phenocopied by the small-molecule inhibitor AZ1. Our results connect viral proteins to human genetic architecture for COVID-19 severity and offer potential therapeutic targets.

## Abstract (web summary)

A SARS-CoV-2-human protein contactome map uncovers an inhibitor of viral replication.

## Main

**Fig. 1:**
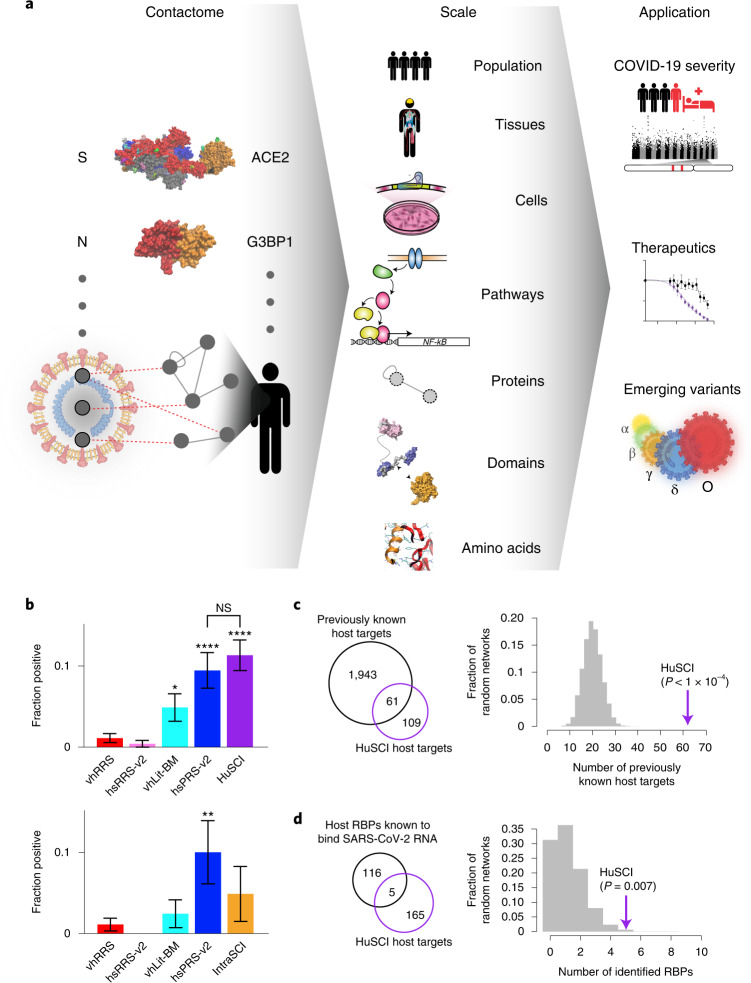
Generation and quality assessment of HuSCI. **a**, The contactome, the sum of physical contacts between viral and host macromolecules, mediates cellular perturbations that enable viral replication and cause disease manifestations. N, nucleocapsid protein; S, spike protein. **b**, Orthogonal validation; fraction of pairs that are yN2H-positive in HuSCI (top, *n* = 282 pair configurations representing 148 HuSCIs interaction pairs) and IntraSCI (bottom, *n* = 41 pair configurations for 25 IntraSCI interaction pairs), in the benchmark positive control sets hsPRS-v2 (*n* = 180 pair configurations for 60 interaction pairs) and vhLit-BM (*n* = 164 pair configurations for 40 interaction pairs), and negative control sets hsRRS-v2 (*n* = 234 pair configurations for 78 protein pairs) and vhRRS (*n* = 360 pair configurations for 178 protein pairs). Asterisks indicate significant differences from vhRRS benchmark (*P = 0.023; **P = 0.005; ****P = 1.57 × 10^−5^ hsPRS-v2, *P* = 1.02 × 10^−7^ HuSCI; NS, not significant; two-sided Fisher’s exact test; center, proportion of positives; error bars, standard error of proportion). Precise *P* values for all dataset pairs, biological repeats and *n* for each test are shown in Supplementary Table 3. **c**, Overlap of SARS-CoV-2 targets identified in HuSCI with previously identified target proteins of other viruses (left) and actual overlap (arrow) compared to *n* = 10,000 randomized control networks (right) (one-sided, empirical *P* < 0.0001). **d**, Host targets identified in HuSCI overlap with RNA-binding proteins (RBPs) bound to SARS-CoV-2 RNA upon infection (left) and actual overlap (arrow) compared to *n* = 10,000 randomized control networks (right) (one-sided, empirical *P* = 0.007).

Despite over 200,000 SARS-CoV-2 publications in the past two years, fundamental questions remain about the molecular mechanisms of genetic risk factors for severe and fatal COVID-19, the cause of long-persisting disease symptoms (long COVID) and the challenge to identify therapeutic targets^1^. These issues remain urgent in light of incomplete vaccination rates, continuously emerging variants and anticipated future pathogens. Fundamentally, infections are initiated by physical contacts between viral proteins and cellular receptors that set off molecular rearrangements culminating in viral entry and unpacking, followed by cellular reprogramming and host defense response triggering. Each of these steps is mediated by contacts between viral and host molecules that determine functional consequences, including proteolytic cleavage or inflammatory signaling, and ultimately clinical manifestations (Fig. 1a). Therefore, understanding the mechanisms by which human genetic variation affects COVID-19, as well as the behavior of newly emerging virus variants such as Delta (𝛿) and Omicron (𝜊), requires knowledge of these contacts to enable studies on how variants functionally alter virus–host interactions. For SARS-CoV-2, the contacts between the viral spike and human ACE2 proteins are documented by several hundred structures. In contrast, no direct interaction partners are known for many other viral proteins, precluding even domain-level contact models. Because co-complex assays predominantly detect indirect protein-associations^2^, the virus–host contactome remains largely unexplored and unknown. To address this fundamental research gap, we systematically identified protein–protein contacts between SARS-CoV-2 and the human proteome.

## Results

### SARS-CoV-2–host contactome mapping

**Extended Data Fig. 1:**
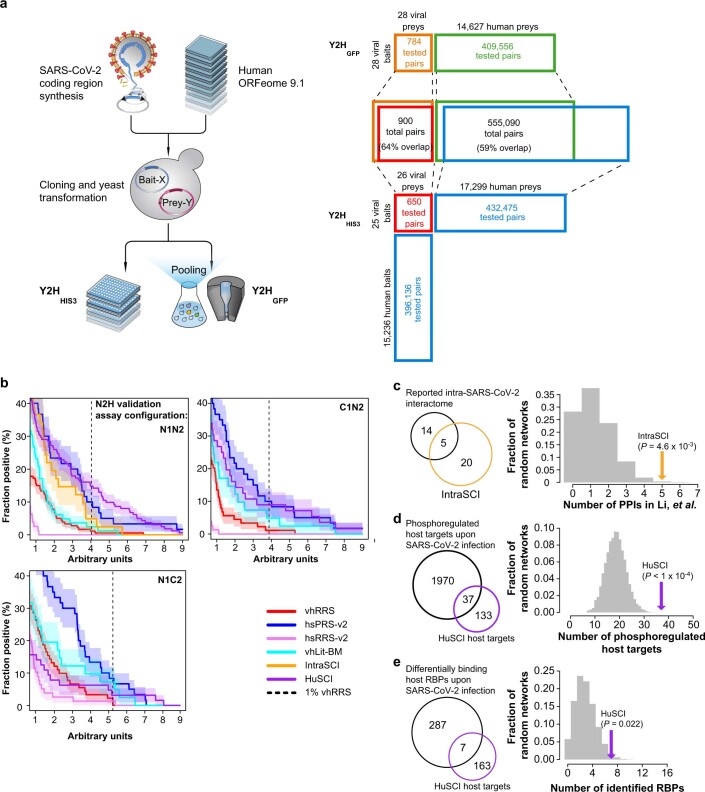
Screening space, orthogonal validation and comparison of HuSCI and IntraSCI to previous SARS-CoV-2 related datasets. **a**, Schematic of the experimental contactome mapping pipelines (left) and screening space for each of the two parallel Y2H_HIS_ and Y2H_GFP_ screens (right). Proportional overlap is given relative to the union of protein pairs tested by both methods. **b**, Rate at which interactions are detected by yN2H for HuSCI and IntraSCI, as well as positive (hsPRS-v2 and vhLit-BM) and negative (hsRRS-v2 and vhRRS) benchmark sets, across stringency thresholds, error band: standard error of proportion. **c**, Left: overlap of previously identified intraviral SARS-CoV-2 interactions and IntraSCI; right: actual overlap (arrow) compared to n = 10,000 randomized control networks. One-sided, empirical *P* = 0.0046. **d**, Left: overlap of host targets identified in HuSCI and differentially phosphorylated proteins following infection by SARS-CoV-2; right: actual overlap (arrow) compared to n = 10,000 randomized control networks. One-sided, empirical *P* < 0.0001. **e**, Left: overlap of host targets identified in HuSCI and RNA Binding Proteins (RBPs) demonstrating differential RNA binding upon SARS-CoV-2 infection; right: actual overlap (arrow) compared to n = 10,000 randomized control networks. One-sided, empirical *P* = 0.022.

We used a multiassay screening and evaluation framework to generate a high-quality virus–host contactome map^2,3^. To increase detection sensitivity in the initial screening by yeast two hybrid (Y2H), we used two complementary assay versions (Extended Data Fig. 1a): (1) a plate-based version using ‘bait’ and ‘prey’ N-terminal fusion proteins encoded on low-copy plasmids and *GAL1-HIS3*-based growth selection (Y2H_HIS3_)^2,3^, and (2) a new system based on the Barcode Fusion Genetics (BFG)-Y2H technology^4^, using a C-terminal fusion prey protein encoded from a high-copy plasmid and selecting cells expressing green fluorescent protein (*GAL1-GFP*) from a pooled liquid culture ﻿(Y2H_GFP_) (unpublished). Using Y2H_HIS3_, 26 viral open reading frames (vORFs; Supplementary Table 1) were screened against 17,472 human ORFs (covering 83% of all pairings of human and viral protein-coding genes, that is 83% ‘search space completeness’) in both orientations; that is, as bait and prey (Extended Data Fig. 1a). Human candidate interactors were pairwise retested in triplicate against every vORF, yielding 118 interactions involving 14 viral and 92 human proteins. We refer to this Y2H_HIS3_-based human SARS-CoV-2 interactome dataset as HuSCI_HIS3_. Using Y2H_GFP_, 28 vORFs were screened against 14,627 human ORFs (70% completeness) (Extended Data Fig. 1a). After stringent filtering and *HIS3*-based verification, this yielded 93 interactions involving 13 viral and 84 human host proteins. We refer to this dataset as HuSCI_GFP_ and to the union with HuSCI_HIS3_ as HuSCI (Supplementary Table 1). We also carried out a targeted screen with previously identified SARS-CoV-1 host interactors; of the 62 testable orthologous SARS-CoV-2–human pairs, six were found to interact (HuSCI_ORTH_) (Supplementary Table 2). Y2H_GFP_ also yielded an intraviral SARS-CoV-2 interactome of 25 binary interactions among 19 vORFs (IntraSCI; Supplementary Table 1). Having collectively identified a contactome of 204 direct virus–host and 25 intraviral interactions among 170 host and 19 viral proteins, we next assessed data quality.

Seven interactions were identified in both HuSCI_GFP_ and HuSCI_HIS3_. Albeit nominally low, this overlap is consistent with the complementary nature of the assays and pipelines. Specifically, the screens interrogated incompletely overlapping protein sets and were each 50%–60% saturated. Each version used for screening has an assay sensitivity of 20%–25%^5^ (fraction of detectable interactions); thus, the overlap is consistent with known screening parameters^2^ and a low false-discovery rate. Moreover, from these parameters we can estimate that HuSCI covers 15%–22% of the complete contactome between SARS-CoV-2 and host proteins (Methods).

To further assess data quality experimentally, we compared detection rates of our datasets in the yeast-based nanoluciferase complementation assay (yN2H)^6^ to those of established human positive and random reference sets (hsPRS-v2 and hsRRS-v2)^5,6^. As additional benchmarks, we derived a set of 55 well-documented binary interactions between human and coronavirus proteins from the curated literature (virus–host literature binary multiple reference set; vhLit-BM) and a virus–host random reference set (vhRRS) (Supplementary Table 3). We tested HuSCI, IntraSCI and each benchmark set by yN2H (Fig. 1b and Extended Data Fig. 1b). At a stringent scoring threshold of 1% vhRRS, the validation rates of both HuSCI alone and the union of HuSCI with IntraSCI (UnionSCI) were statistically indistinguishable from the two positive control sets (hsPRS-v2, *P* = 0.76; vhLit-BM, *P* = 0.06; Fisher’s exact test versus UnionSCI), and each was significantly higher than those of the negative control sets (hsRRS-v2, *P* = 4 × 10^−7^; vhRRS, *P* = 1 × 10^−7^; Fisher’s exact test versus UnionSCI; Fig. 1b and Supplementary Table 3). Thus, the biophysical quality of our virus–host contactome map is at least on par with high-quality interactions supported by multiple experiments in the curated literature. Although IntraSCI is too small for a separate evaluation by yN2H, 5 of 25 interactions overlap with a previous study^7^ (*P* = 4.6 × 10^−3^, empirical test; Extended Data Fig. 1c).

**Fig. 2:**
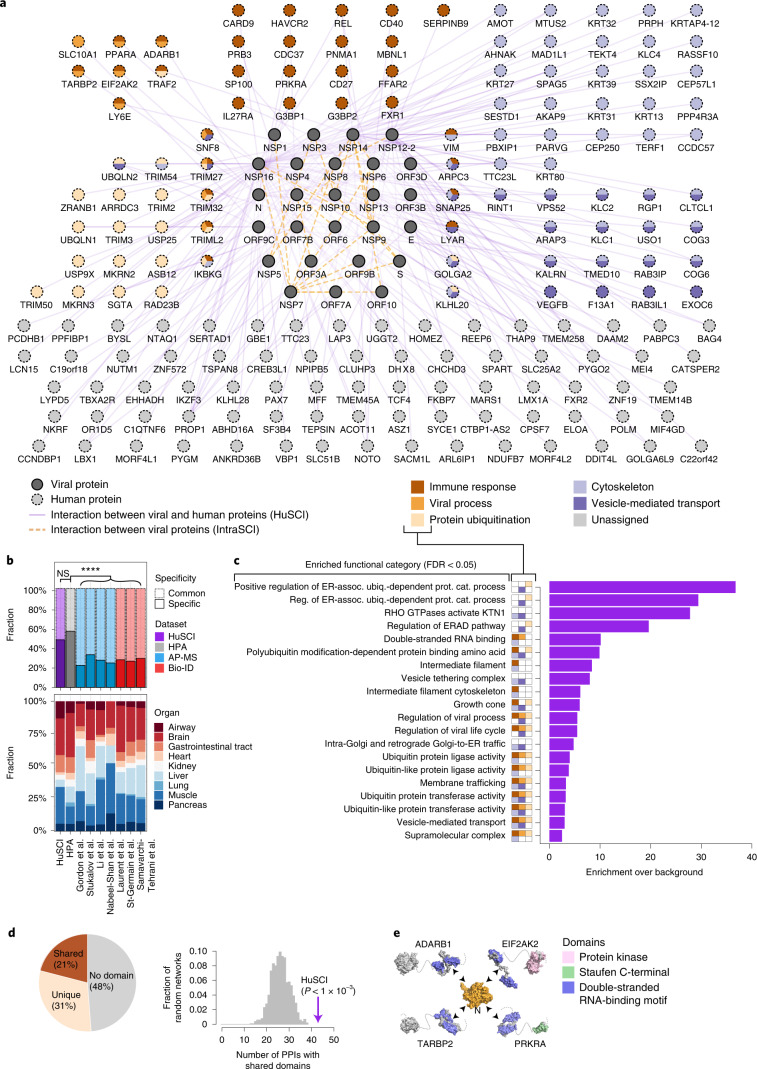
Network representation and functional assessment of HuSCI. **a**, Combined HuSCI and IntraSCI networks. Node colors of human proteins represent broad enriched functions as indicated in legend. Node labels for human proteins correspond to approved HGNC symbols; accession identifiers and descriptions are listed in Supplementary Table 1. **b**, Proportion of host targets in common and specific expression groups in all (top) and in SARS-CoV-2 RNA-positive organs (bottom) across eight datasets: purple, HuSCI; gray, HPA^21^; blue, AP-MS datasets from Gordon et al.^12,13^, Stukalov et al.^9^, Li et al.^14^ and Nabeel-Shah et al.^15^; red, BioID datasets from Laurent et al.^16^, St-Germain et al.^17^ and Samavarchi-Tehrani et al.^18^. Two-sided Fisher’s exact test, Bonferroni adjusted *P* < 0.0001. Full statistical details and exact *P* values are listed in Supplementary Table 4. **c**, Functions enriched among host proteins found in HuSCI (*P* = 0.05, Fisher’s exact test with FDR correction). Broad functional groups are indicated in small boxes according to legend in panel a. Full statistical details are listed in Supplementary Table 5. ER-assoc. ubiq.-dependent prot. cat., Endoplasmic reticulum-associated ubiquitin-dependent protein catabolic. **d**, Proportion of virus–host interactions in which the human protein has domains that are present in other interactors of the viral protein (shared), not present in other interactors of the viral protein (unique) or no annotated domains (left) and number of shared-domain interactions in HuSCI (arrow) compared to *n* = 1,000 randomized control networks (gray distribution) (right). One-sided empirical *P* < 0.001. PPI, protein-protein interactions. **e**, Exemplary ‘shared-domain interaction’ between the viral nucleocapsid protein and four interactors containing a double-stranded RNA-binding motif. Domain colors according to legend; gray parts lack domain annotations.

The biological relevance of our virus–host contactome map is suggested by the observations that the identified host proteins are enriched for (1) known targets of other viruses^8^ (*P* < 1 × 10^−4^, empirical test; Fig. 1c), (2) proteins that change phosphorylation status upon SARS-CoV-2 infection^9,10^ (*P* < 1 × 10^−4^, empirical test; Extended Data Fig. 1d), (3) proteins that directly interact with SARS-CoV-2 RNA^11^ (*P* = 0.007, empirical test; Fig. 1d) and (4) proteins that change RNA-binding status during SARS-CoV-2 infection^11^ (*P* = 0.022, empirical test; Extended Data Fig. 1e). These results demonstrate that IntraSCI and HuSCI (Fig. 2a) are of high biophysical quality and enriched for host proteins relevant to SARS-CoV-2 biology.

### Complementarity of contactome and co-complex datasets

**Extended Data Fig. 2:**
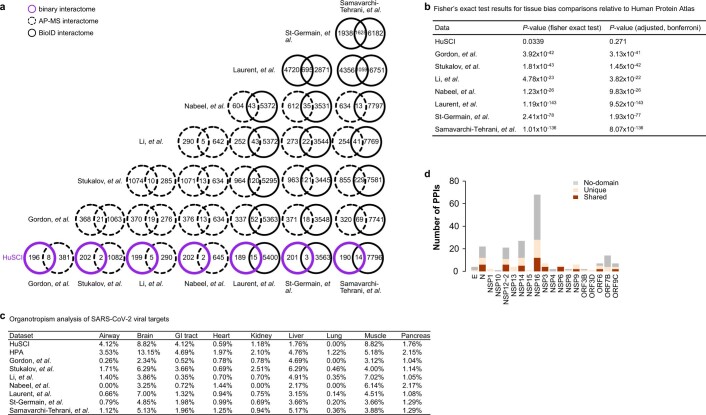
Comparison of HuSCI with SARS-CoV-2 association and proximity datasets. **a**, Overlap of viral-human protein pairs between HuSCI, four AP-MS and three BioID based datasets (Gordon et al.^12,13^, Stukalov et al.^9^, Li et al.^14^, Nabeel-Shah et al.^15^, Laurent et al.^16^, St-Germain et al.^17^, Samavarchi-Tehrani et al.^18^). **b**, Statistical analysis of representation of host targets in common and specific expression groups from datasets in (**a**), compared to the Human Protein Atlas22 (HPA) (Fisher’s exact test with Bonferroni correction). **c**, Organotropism analysis across SARS-CoV-2 infected organs from datasets in (**b**). The percentage of genes within each dataset with specific organotropism (‘tissue-specific’ expression in tissues grouped into organ systems). **b** and **c**, Full analysis is shown in Supplementary Table 4**. d**, Proportion of HuSCI host interactors per SARS-CoV-2 protein in which the human protein has: domains present in other interactors of the viral protein (shared); domains not present in other interactors of the viral protein (unique); no structural domains. Full analysis is shown in Supplementary Table 6.

Previous studies investigating host and SARS-CoV-2 proteins used either affinity purification followed by mass spectrometry (AP-MS) to identify co-complex associations^9,12–15^ or biotin identification (BioID) to find proteins in spatial proximity^16–18^. However, co-complex maps capture largely indirect associations in stable complexes that persist through affinity purification^2^ and, likely due to experimental differences, the datasets exhibit limited agreement among each other (Extended Data Fig. 2a). For a subset of such co-complex associations, contacts can be computationally modeled^19^. In contrast, binary interactome maps provide direct contact partners and are enriched for regulatory interactions^2^. Despite these differences, 20 of the 204 HuSCI-interacting pairs were found in co-complex and BioID studies, and 58 (34%) of the 170 HuSCI host proteins were associated with a SARS-CoV-2 protein by these studies (Supplementary Table 1). Thus, the contactome map is consistent with previous indirect association datasets while providing substantial novelty.

Although SARS-CoV-2 primarily infects lung and airway tissues, it can spread to additional tissues and this expanded tropism is characteristic for COVID-19 and important for long COVID symptoms^20^. As previous SARS-CoV-2 interaction datasets could only detect host proteins expressed in the specific assay cell lines, we wondered whether HuSCI was also complementary in terms of the tissue specificity of identified host proteins. Using the Human Protein Atlas (HPA)^21^, we defined ‘tissue-specific’ and ‘common’ human proteins (Supplementary Table 4). Whereas the AP-MS and BioID data are biased toward common host proteins, HuSCI is more representative of the human proteome and shows good coverage of proteins expressed in the diverse tissues in which SARS-CoV-2 RNA has been detected^22^ (Fig. 2b, Extended Data Fig. 2b,c and Supplementary Table 4). Thus, the HuSCI contactome has unique advantages for understanding tissue-specific perturbations by SARS-CoV-2.

### SARS-CoV-2 targeted functions

To understand which host functions are directly perturbed by the virus, we investigated SARS-CoV-2 targeted pathways. Broad functions enriched among host proteins include (1) immune response, (2) viral process, (3) protein ubiquitination, (4) cytoskeleton and (5) vesicle-mediated transport (Fig. 2c). These largely agree with functions identified in association and proximity datasets^9,12–18^ (Supplementary Table 5). Focusing on immune pathways, we noticed that NSP9, NSP14 and NSP16 contact key regulators of cytokine production such as REL (c-REL proto-oncogene, NF-κB subunit), IKBKG (inhibitor of NF-κB kinase regulatory subunit gamma, also known as IKK𝛾 or NEMO) and TRAF2 (tumor necrosis factor (TNF) receptor-associated factor 2). HuSCI interactors of the membrane-spanning NSP6 were enriched for immune receptors (*P* < 0.01, empirical test), including CD40 and IL27RA (IL27 receptor subunit alpha). Intriguingly, NSP6 also directly interacts with LY6E, a host restriction factor that limits viral entry for SARS-CoV-2 and other coronaviruses^23^. Several other targets are RNA-binding proteins that function in innate immunity and response to viral infection^24^. MKRN2, together with G3BP1/2, has been suggested to regulate olfactory signaling mRNAs^25^, pointing to potential mechanistic links underlying anosmia in COVID-19. Thus, direct SARS-CoV-2 protein interactors function in immune pathways and viral processes relevant to COVID-19.

### Viral proteins contact shared host-protein domains

The restricted size of viral genomes limits their coding potential. We therefore wondered to what extent this limitation yielded viral proteins that bind multiple human proteins via target-shared domains, thus offering opportunities for structure-based drug discovery. We sought domains shared by multiple human targets of each viral protein. In the contactome, SARS-CoV-2 proteins engaged in 43 interactions involving such shared domains (21% of HuSCI; *P* < 0.001, empirical test; Fig. 2d, Extended Data Fig. 2d and Supplementary Table 6), corresponding to 22 significant virus protein-domain pairs (*P* < 0.001, empirical test; Supplementary Table 6). Although the difference was not statistically significant, the 21% proportion of the virus–host contactome with shared-domain interactions was numerically higher than the corresponding 17% in the human reference interactome network (HuRI)^26^. Specific examples in HuSCI include four interactors of the nucleocapsid protein sharing the double-stranded RNA-binding motif (*P* < 0.05, Fisher’s test; Fig. 2e) and the recently confirmed finding that viral nucleocapsid protein interacts with the NTF2 domains of G3BP1 and G3BP2^27^. Disease-causing mutations are located in the interaction interfaces of the enriched domains of several human proteins (for example, TNF receptor domain of CD40 (ref. ^28^) or zf-CCCH in MKRN3 (ref. ^29^)). Thus, recurrent structural themes may reflect binding mechanisms that are subject to modulation by human coding variants affecting infection outcome^30,31^ or by rationally designed drugs.

### HuSCI links to COVID-19 risk loci

**Fig. 3:**
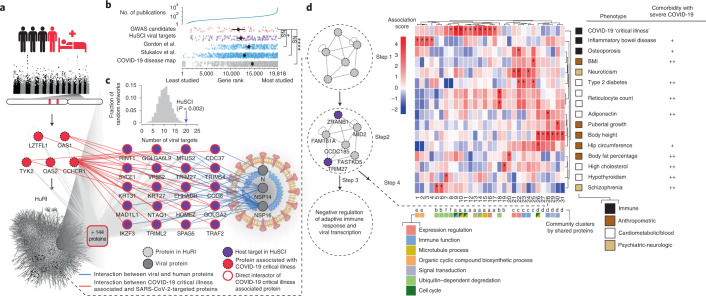
HuSCI host targets link to genetic variation for severe COVID-19. **a**, HuRI interactors (gray nodes) of COVID-19 ‘critical illness proteins’ loci (seed, red nodes) include a significant (*P* = 0.0009, empirical test) number of direct SARS-CoV-2 targeted proteins (purple nodes). A total of 144 additional seed protein interactors are not resolved individually. Node and edge colors according to legend. **b**, Genes in indicated COVID-19 datasets ranked across the human genome by number of publications. Number of publications is indicated by the top panel on log scale. Asterisks indicate significant differences relative to COVID-19-associated genes^32,33^; NS, *P* = 1 (HuSCI) and *P* = 0.36 (Stuckalov et al.); *P = 0.047; ****P = 0.000014, two-sided Mann–Whitney *U* test, Bonferroni correction, from top to bottom: *n* = 45, 170, 383, 876 and 849; error bars are 95% confidence intervals of the mean, calculated by 1,000 bootstrap samples. **c**, Virus-interactor enrichment: number of direct SARS-CoV-2 protein interacting HuSCI proteins in the subnetwork formed by proteins encoded by seed proteins^32,33^ and their first-level interactors (arrow) compared to *n* = 10,000 randomized control networks (gray distribution). **d**, Of 3,603 communities in Human Reference Interactome (HuRI) with ≥4 members (step 1), 204 are significantly targeted by SARS-CoV-2 (two-sided nominal *P* < 0.05; Fisher’s exact test) (step 2); Gene Ontology (GO) enrichment identifies functions associated with each community (step 3); and MAGMA identifies 31 communities significantly associated with human traits (FDR < 0.05) (step 4), the great majority of which are COVID-19 comorbidities. Example community 28 is significantly targeted by SARS-CoV-2 in HuSCI (two-sided *P* = 0.0078; Fisher’s exact test, uncorrected) and enriched for negative regulation of adaptive immune response and viral transcription. Functional descriptors in squared boxes according to legend (Supplementary Table 8); relation of indicated traits to COVID-19 is indicated in rightmost column as general link (+) (e.g., via immunity) and clinical evidence for modulation of diseases symptoms and risk for severe or long COVID ( + + ; Extended Data Fig. 3f). BMI, body mass index.

**Extended Data Fig. 3:**
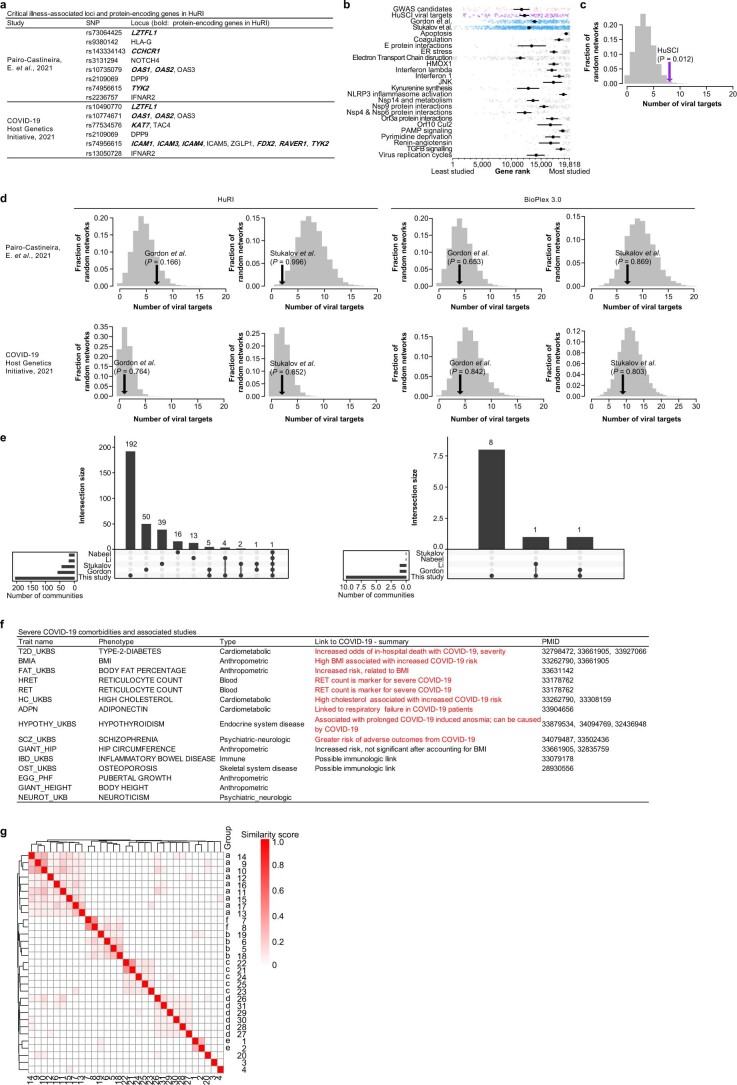
Traits associated with COVID-19 severity. **a**, Table showing COVID-19 critical illness associated loci from two GWAS meta-analyses^32,33^. Locus-associated proteins present in HuRI are marked in bold. **b**, Genes in indicated COVID-19 datasets ranked across the human genome by number of publications. Error bars are 95% confidence intervals of the mean, calculated by 1,000 bootstrap samples (from top to bottom n = 45, 170, 383, 876, 29, 75, 25, 71, 233, 46, 49, 45, 15, 33, 10, 71, 97, 47, 58, 9, 46, 22, 20, 23, 39). **c**, Virus-interactor enrichment in contactome: number of direct SARS-CoV-2 protein interacting HuSCI proteins in a HuRI subnetwork formed by proteins encoded by COVID-19 critical illness associated loci^33^ (marked in bold in table (**a**)) and their first level interactors (arrow) compared to n = 10,000 randomized control networks (gray distribution). One-sided, empirical *P* = 0.012. **d**, Virus-interactor enrichment in co-complex associations: number of SARS-CoV-2 associated human proteins in two AP-MS based studies^9,13^ in a subnetwork formed by proteins encoded by COVID-19 critical illness associated loci^32,33^ (marked in bold in table in (**a**)) and their first level interactors (arrow) either in HuRI or BioPlex 3.0. The comparisons are against n = 10,000 randomized control networks (gray distribution). One-sided, empirical *P* values are shown for each dataset. **e**, Upset plots showing number of communities targeted by SARS-CoV-2 (left) and associated with severe COVID-19 (right) in HuSCI and AP-MS based datasets. **f**, Table showing 15 traits for genetic variation identified within targeted network communities. An association with severe COVID-19 comorbidities is indicated, as well as trait references: T2D_UKBS^91–93^, BMIA^92,94^, FAT_UKBS^95^, HRET^96^, RET^96^, HC_UKBS^94,97^, ADPN^98^, HYPOTHY_UKBS^43,99,100^, SCZ_UKBS^44,101^, GIANT_HIP^92,102^, IBD_UKBS^103^, OST_UKBS^104^, EGG_PHF, GIANT_HEIGHT, NEUROT_UKB. **g**, Grouping of 31 network communities with significantly associated traits shown in Fig. 3d by protein membership measured by Jaccard similarity according to legend.

The severity of COVID-19 symptoms and outcomes are highly variable, and understanding the underlying molecular mechanisms may enable effective treatments. Recently, two independent meta-studies identified genetic loci that are associated with severe COVID-19 illness^32,33^ (Fig. 3a and Extended Data Fig. 3a), but mechanistic links to viral infection remain unknown. Similarly, several preconditions increase the risk of severe COVID-19, but for these, the molecular links are also poorly understood. At least two models can help to conceptualize how this genetic variation relates to virally targeted host proteins. In a ‘direct’ model, genetic variation in targeted host proteins modulates disease outcome, exemplified by the interaction of adenovirus E1A oncoprotein with the tumor suppressor protein pRb^34^. In an alternative ‘indirect’ model, genetic variation in the network neighborhood of targeted host proteins modulates the downstream effects and thereby influences disease outcome. A precedent for this model was observed in a plant system, where pathogen-targeted host proteins tend to interact with proteins relevant to disease severity and fitness (encoded by highly variable genes under balancing selection)^35^. The availability of a high-quality contactome map enabled us to address this fundamental question for COVID-19. Because bias toward well-studied proteins in the SARS-CoV-2 literature^36^ (Fig. 3b and Extended Data Fig. 3b) limits mechanistic understanding and can cause artifacts, we focused our analyses on systematic protein interaction datasets. The direct model was not supported, given that no targeted host protein from HuSCI was encoded from a critical illness associated locus^32,33^ (‘critical illness proteins’), and only one (HLA-G, associated with ORF3) was found in a single co-complex study^9^. Investigating the indirect model, we sought contacts between targeted host proteins and critical illness proteins, finding 20 (*P* = 0.002, empirical test; Fig. 3c)^32^ and 8 (*P* = 0.012, empirical test; Extended Data Fig. 3a, c)^33^ in the binary HuRI host network map. In contrast, the virus-associated host-protein sets from AP-MS studies^9,12,13^ interact with no more critical illness proteins than expected by chance (Extended Data Fig. 3d). Functionally, the HuSCI host-target proteins linking critical illness to SARS-CoV-2 proteins are enriched in microtubule organization, membrane trafficking and TNF signaling annotations (Supplementary Table 7). Intriguingly, three of seven direct OAS1 interactors are targeted by NSP14 and NSP16, and all three have Golgi- and membrane trafficking-related functions, providing protein contacts that support the finding that the Neanderthal-derived protective OAS1 variant promotes degradation of viral RNA in endoplasmic reticulum- and Golgi-derived virus replication organelles^37^. These observations indicate that, consistent with the indirect model, clinically relevant genetic variation acts in the local network neighborhood of viral contact proteins.

To further explore the local subnetworks surrounding targeted host proteins and their links to human genetic variation, we identified 204 subnetwork communities in HuRI^26^ (Fig. 3d) that were significantly targeted by SARS-CoV-2 (nominal *P* < 0.05, Fisher’s exact test; Supplementary Table 8). Examples include community 28, enriched for ‘negative regulation of viral transcription’ (false discovery rate (FDR) = 0.0018; Fig. 3d) and community 52, enriched for ‘Arp2/3 complex-mediated actin nucleation’ (FDR = 0.0002; Supplementary Table 8). The Arp2/3 complex enables human respiratory viruses to spread among adjacent cells without forming virions^38^, and ARPC3 scored among the top 50 in two CRISPR screens for SARS-CoV-2 host factors^39,40^. We then asked whether direct viral target proteins and proteins in each community are encoded by genes associated with human traits of 114 uniformly processed genome-wide association studies (GWASs)^41^. Variation in genes encoding direct viral targets was only associated with ‘depression’ (FDR = 0.03, MAGMA). In contrast, among the communities, genetic variation associated with severe COVID-19 illness was associated with ten virus-targeted communities, more communities than any other human trait. In contrast, host-protein sets from AP-MS studies were enriched in fewer communities (nominal *P* < 0.05, Fisher’s exact test; Extended Data Fig. 3e and Supplementary Table 8), and only one host-protein-enriched community each from two AP-MS datasets was enriched for genetic variation associated with severe COVID-19 (refs. ^13,14^) (Li et al. community 14 and Gordon et al. community 11; Extended Data Fig. 3f). Intriguingly, of the 14 human traits (from 15 studies) associated with 20 additional HuSCI-target-enriched communities, 8 traits (from 9 studies) are clinical risk factors for severe COVID-19 and long COVID, including high body mass index (BMI)^42^, hypothyroidism^43^ and schizophrenia^44^ (*P* = 0.01, Fisher’s exact test; Fig. 3d, Extended Data Fig. 3e,f and Supplementary Table 8). These links between viral targets and genetic variation associated with COVID-19 comorbidities open the possibility that this genetic variation may impact the course of infection and severity of COVID-19 independent of trait manifestation. Other traits associated with host-target-enriched communities, such as neuroticism, have not been linked to COVID-19 symptoms, possibly because the genetic influence is masked by confounding parameters such as behavior^45^, and should be considered in the future. Together, these results suggest that the HuSCI contactome map is a powerful and unique resource for studying molecular mechanisms by which human genetics affect the outcome of SARS-CoV-2 infection.

### Validation of pathways and host targets

**Fig. 4:**
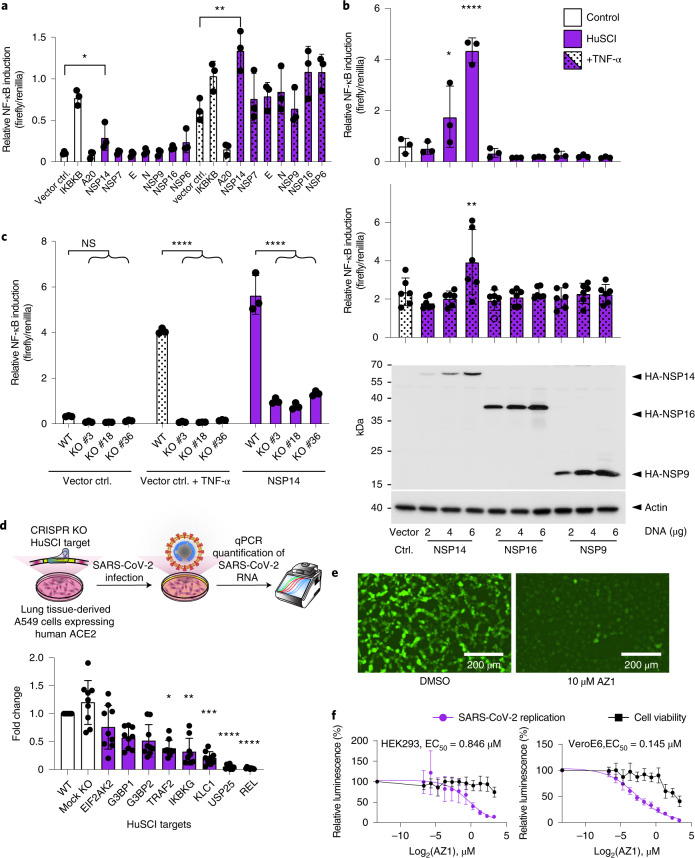
Validation of pathways and host targets. **a**, Relative NF-κB transcriptional reporter activity in unstimulated (left) and TNF-α-stimulated conditions (one-way analysis of variance (ANOVA) with Dunnett’s multiple comparisons test, *P* = 0.0395 and *P* = 0.0047, respectively). Error bars represent standard deviation of the mean, *n* = 3. **b**, Relative NF-κB transcriptional reporter activity at different amounts of transfected viral protein-encoding plasmid in unstimulated (top) and TNF-α-stimulated conditions (middle) (one-way ANOVA with Dunnett’s multiple comparisons test: *P = 0.0183, ****P < 0.0001 and **P = 0.0012, respectively). Error bars represent standard deviation of the mean, *n* = 3 (top) and *n* = 6 (middle). Representative anti-hemagglutinin (HA) western blot demonstrating levels of tagged viral protein in titration experiments relative to actin beta (ACTB) loading controls (bottom). **a** and **b**, Precise *P* values, biological repeats and *n* for each test are shown in Extended Data Fig. 4 and Supplementary Table 9. **c**, Relative NF-κB transcriptional reporter activity under unstimulated (left), TNF-α-stimulated (middle) and NSP14-induced conditions in wild-type (WT) and three independent IKBKG KO clones of HEK293 cells (two-way ANOVA with Dunnett’s multiple comparisons test). Error bars represent standard deviation of the mean, *n* = 3. Precise *P* values, biological repeats and *n* for each test are shown in Extended Data Fig. 4. ctrl., control. **d**, Schematic of viral replication assay (top) and viral replication in wild-type, mock KO and CRISPR KOs of the indicated HuSCI host targets (bottom) (Kruskal–Wallis with Dunn’s multiple comparisons test, * *P* = 0.031, ** *P* = 0.0047, *** *P* = 0.0003, **** *P* < 0.0001, respectively). Error bars represent standard deviation of the mean, *n* = 9. Precise *P* values, biological repeats and *n* for each test are shown in Extended Data Fig. 4 and Supplementary Table 10. **e**, Fluorescence microscopy images showing replication of icSARS-CoV-2-mNeonGreen in infected Vero E6 cells treated with 10 µM AZ1 or solvent (DMSO, dimethylsulfoxide). **f**, Cell viability and relative replication of icSARS-CoV-2-nanoluciferase in HEK293 cells (left) and Vero E6 cells (right) at different concentrations of AZ1. The EC_50_ values were calculated with a variable slope model. Error bars represent standard deviation of the mean, *n* = 8 biological repeats and full analysis in Supplementary Table 11. Source data

**Extended Data Fig. 4:**
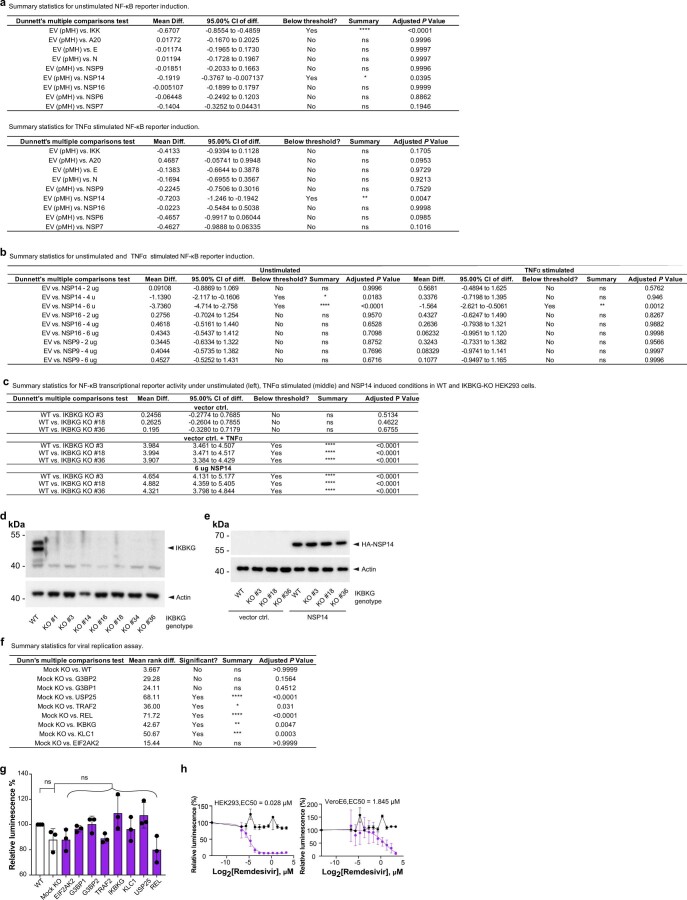
Effect of viral proteins on NF-κB reporter activity and of viral interactors on viral replication. **a**, Tables showing statistical details of NF-κB transcriptional reporter activity in the absence and presence of selected viral proteins under unstimulated (top) and TNFα stimulated (bottom) conditions. One-way ANOVA with Dunnett’s multiple comparisons test, n = 3, adjusted *P* values are shown. **b**, Table showing statistical details of NF-κB transcriptional reporter activity at different amounts of transfected viral protein-encoded plasmid under unstimulated (left) and TNFα stimulated conditions (right). One-way ANOVA with Dunnett’s multiple comparisons test, n = 3 and n = 6, respectively, adjusted *P* values are shown. **a** and **b**, Raw data and full analysis is shown in Supplementary Table 9. **c**, Table showing statistical details of NF-κB transcriptional reporter activity under unstimulated (left), TNFα-stimulated (middle) and NSP14-induced conditions in WT and IKBKG KO HEK293 cells (two-way ANOVA with Dunnett’s multiple comparisons test, n = 3), adjusted *P* values are shown. **d**, Representative anti-IKBKG (top) western blot demonstrating levels of IKBKG in WT and three independent IKBKG knockout clones of HEK293 cells relative to actin beta (ACTB) loading controls (bottom). **e**, Representative anti-hemagglutinin (HA) western blot demonstrating levels of tagged NSP14 protein in NF-κB induction experiments relative to actin beta (ACTB) loading controls (bottom). **f**, Table showing statistical details of viral replication in wild-type, mock KO and CRISPR KOs of the indicated HuSCI host proteins. Kruskal-Wallis with Dunn’s multiple comparisons test, n = 9. Adjusted *P* values are shown. **g**, Cell viability of mock KO and CRISPR KOs of the indicated HuSCI host proteins relative to WT cells. Kruskal-Wallis with Dunn’s multiple comparisons test, n = 3. Adjusted, Fisher’s exact *P* values are shown. **f** and **g**, Raw data, Fisher’s exact *P* values, and full analysis is shown in Supplementary Table 10. **h**, Cell viability and relative replication of icSARS-CoV-2-nanoluciferase in HEK293 cells (left) and Vero E6 cells (right) at different concentrations of remdesivir. The EC50 values shown for each cell line were calculated with a variable slope model. Error bars: standard deviation of the mean, n = 3 biological repeats, full analysis in Supplementary Table 11. Source data

We next explored specific hypotheses for viral proteins and human target functions. Both literature reports and our analyses suggest a role for NF-κB immune signaling in SARS-CoV-2 infection. Because we observed multiple interactions of viral proteins with different members of the NF-κB signaling pathway, we used reporter assays to determine whether and in which direction (that is, activating or inhibiting) viral factors modulate pathway activity. Transfection of NSP14, which interacts with multiple positive NF-κB regulators, resulted in dose-dependent transcriptional activation of NF-κB and even further augmented NF-κB activity following proinflammatory TNF-α stimulation in HEK293 cells (Fig. 4a,b, Extended Data Fig. 4a,b and Supplementary Table 9). This finding suggests that SARS-CoV-2 can induce a proinflammatory state during COVID-19 via direct interaction of NSP14 with NF-κB activators. These results are corroborated by a study that implicates IMPDH2 in NF-κB pathway activation by NSP14 (ref. ^46^). Moreover, transcriptional profiling experiments have demonstrated NF-κB activation in HEK293 cells and in patients following SARS-CoV-2 infection^47,48^. As TNF-α has a central role in the cytokine storm that contributes to many COVID-19 deaths^49^, the observation that SARS-CoV-2 activates this system in a cell-intrinsic manner may have therapeutic implications.

We explored the role of the NSP14 interactor IKBKG/NEMO, an essential mediator of canonical NF-κB signaling^50^, for transcriptional activation. We generated IKBKG HEK293 knockout (KO) clones (Extended Data Fig. 4) and checked for NF-κB activation in three independent clones after NSP14 transfection (Fig. 4c). IKBKG deficiency abolished NF-κB activation in response to TNF-α and severely impaired NSP14-induced NF-κB activation, providing evidence for a functional role of IKBKG in driving NF-κB activation by NSP14. Interestingly, the residual NF-κB reporter induction upon NSP14 expression in the KO cells indicates that other NSP14 interactors (for example, TRAF2 and REL) contribute to the full NF-κB transcriptional response.

We wondered whether NF-κB signaling proteins and virally targeted host proteins in enriched functional groups other than ‘immune response’ (Fig. 2a) are important for viral replication. After generating A549 alveolar basal epithelial adenocarcinoma cells that exogenously express human ACE2 (A549-ACE2), we quantified viral replication in the presence and absence of CRISPR-Cas9-mediated KO of viral-target-encoding genes. Of eight genes that were selected from enriched functional groups and successfully knocked out, deletion of five (63%) resulted in a significant decrease of viral replication (Fig. 4d). Intriguingly, deletion of three NSP14-interacting proteins of the NF-κB signaling system (REL, IKBKG and TRAF2) resulted in strong reduction of viral replication (Fig. 4d and Extended Data Fig. 4f,g). This finding is consistent with a model in which SARS-CoV-2 directly activates NF-κB via NSP14, with this activation being required for successful viral replication. Deletion of kinesin light chain 1 (KLC1), a cargo adaptor protein for microtubule mediated transport, caused reduction of replication by ~80% (*P* < 0.0001, Kruskal–Wallis test). Beyond this observation, deletion of ubiquitin-specific peptidase 25 (USP25), which has antiviral functions in influenza and herpes infections^51^, resulted in essentially complete elimination of viral replication without impacting cell growth, suggesting that human USP25 is required by SARS-CoV-2 (Fig. 4d, Extended Data Fig. 4f,g and Supplementary Table 10).

Inspired by the strong effect on viral replication, we explored USP25 as an antiviral drug target using the small molecule AZ1, which effectively inhibits USP25 and USP28 enzymatic activity^52^. Using an infectious clone-derived SARS-CoV-2 (icSARS-COV-2) harboring a mNeonGreen marker^53^, we showed that treatment with 10 µM AZ1 effectively inhibits SARS-CoV-2 replication in Vero E6 cells (Fig. 4e). Next, we used an independent icSARS-CoV-2 expressing nanoluciferase^54^ for dose titrations. The AZ1 compound interfered with SARS-CoV-2 replication with half-maximum effective concentration (EC_50_) values of 0.8 µM and 0.1 µM in HEK293-ACE2 and Vero E6 cells, respectively (Fig. 4f and Supplementary Table 11), on par with the effects of the clinically approved remdesivir (Extended Data Fig. 4h). Effective concentrations are in the range of the half-maximal inhibitory concentration determined for inhibition of USP25/28 enzymatic activities^52^, further supporting that USP25 is necessary for SARS-CoV-2 replication. Although the antiviral activity of AZ1 was independently identified in a small-molecule screen^55^, our results inform mechanistic studies by identifying NSP16 as a viral interaction partner. NSP16 and associated complexes methylate viral RNA to prevent its detection and destruction by the innate immune system^56,57^. The stable recruitment of USP25 may protect this complex from ubiquitination and degradation by the host defense machinery. Although elucidating precise mechanisms will require further studies, these findings illustrate the high potential of the HuSCI contactome map in helping to understand and inhibit the SARS-CoV-2 life cycle.

### Perturbed contactome in SARS-CoV-2 variants

**Extended Data Fig. 5:**
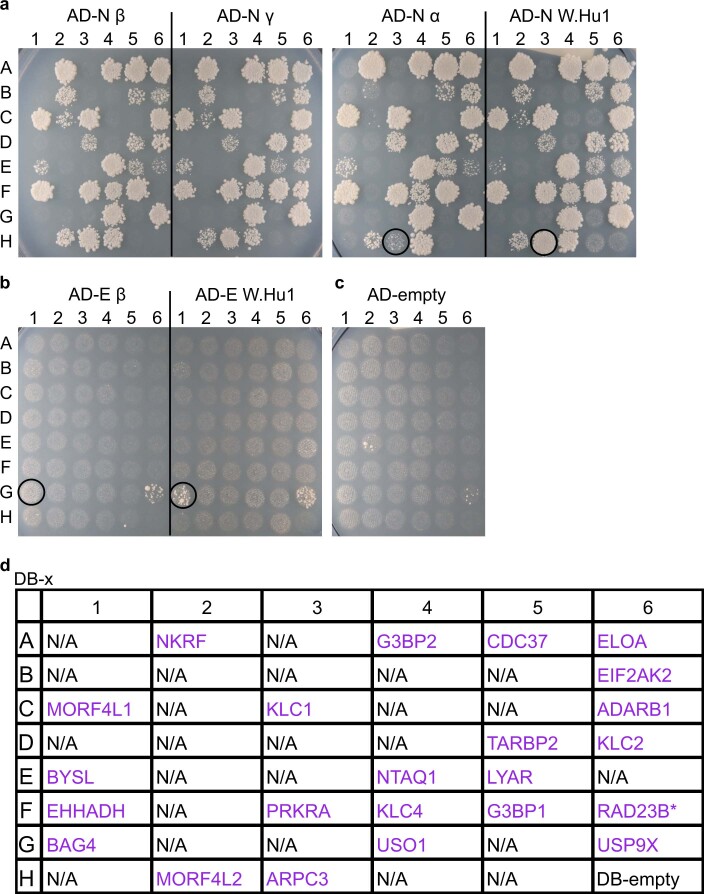
Mutations of SARS-CoV-2 variants affect specific interactions with uSCI host targets. **a**, Y2H_HIS3_ yeast growth on selective plates of HuSCI interaction partners as DB-fusion proteins tested against AD-fusion of the SARS-CoV-2 Nucleocapsid (AD-N) protein (Wuhan-Hu1, original screen) and AD-N containing ‘lineage defining’ amino acid substitutions: D3L and S235F (α-strain), T205I (β-strain), or P80R (γ-strain). Shown is one representative result of 5 repeats. **b**, Y2H_HIS3_ yeast growth on selective plates of HuSCI interaction partners as DB-fusion proteins tested against AD-fusion of the SARS-CoV-2 Envelope (AD-E) protein (Wuhan-Hu1, original screen) or AD-E containing ‘lineage defining’ substitution P71L (β-strain). Shown is one representative Y2H_HIS3_ result on selective media, out of 2 repeats. **a** - **c**, Black circles indicate changes in yeast colony growth between human proteins tested against viral variant ORFs or the originally screened Wuhan strain ORFs observed consistently across all repeats. **c**, AD-empty control plate for **a, b** indicates lack of autoactivation. **d**, Layout of DB-fusion HuSCI interactors (purple) tested with AD fusion SARS-CoV-2 proteins or AD-empty control, respectively in **a - c**. N/A indicates human interactors.

Evaluating the impact of novel viral strains on the contactome has been largely restricted to spike protein interactions with ACE2 and antibodies^58^. Wondering if coding variants in other viral proteins perturb the contactome and thereby modulate viral effects, we explored the potential of 19 SARS-CoV-2 mutations in 14 variants of 9 proteins from the Alpha, Beta, Gamma and Delta strains to alter interactions with host contact targets in HuSCI (Supplementary Table 12). Indeed, some mutations resulted in perturbed interactions. The Alpha strain mutant combination D3L, S235F in the nucleocapsid protein reduced interaction with ARPC3, the SARS-CoV-2 host factor discussed above. Similarly, the Beta-strain mutation P71L in the envelope (E) protein diminished the interaction with BAG4, an antiapoptotic protein involved in TNF signaling (Extended Data Fig. 5). Although it is currently unknown whether the respective interactions promote viral replication or facilitate immune recognition, the observed changes demonstrate the plasticity of the contactome and, together with recent reports of increased replication of the Delta strain^59^, strongly suggest that this dimension of viral evolution should also be monitored to assess the risk posed by emerging variants.

## Discussion

In summary, we present a validated contactome map, HuSCI, which provides direct interactions between SARS-CoV-2 and human target proteins in pathways and tissues relevant to COVID-19. HuSCI enables identification of paths of direct contact between viral target proteins and proteins encoded from loci that modify the risk for critical COVID-19 illness and important comorbidities. Examining specific hypotheses for both viral and host proteins, we demonstrate that NSP14 activates the NF-κB pathway even beyond pathway activation by cytokines. Moreover, the majority of the virally targeted host proteins we evaluated, including key NF-κB regulators, are required for efficient SARS-CoV-2 replication. For one of these targeted host proteins, USP25, we confirm that a small-molecule inhibitor can dramatically reduce viral replication and implicate a mechanism for this potential therapeutic. Last, we demonstrate that coding changes in SARS-CoV-2 strains perturb the intracellular interactome. We anticipate that these findings and the contactome resource will stimulate important research toward characterizing new viral strains, understanding the mechanism of COVID-19 symptoms and developing therapies for current and future pandemics.

## Methods

### Cloning SARS-CoV-2 ORFs

Two independent SARS-CoV-2 vORF collections were constructed in Gateway entry vectors. The Y2H_GFP_ collection^60^ includes all but one (NSP11 was too short for Gateway cloning) codon-optimized ORF of SARS-CoV-2, synthesized based on a published genome^61^, which were cloned with and without stop codon, to enable C-terminal fusions. The Y2H_HIS3_ entry clone collection is based on National Center for Biotechnology Information (NCBI) accession number NC_045512.2 and annotation^62^. Y2H_HIS3_ vORFs were synthesized by Twist Bioscience without codon optimization and included 5´ and 3´ linkers with SfiI restriction sites. The 5´ linker incorporates a translational start ATG flanked by BamHI sites; the 3´ linker provides a stop codon flanked by PacI and AsiSI restriction sites. For Y2H_HIS3_, vORFs were cloned into pENTR223.1 using SfiI restriction cloning, and the alternative ATG was removed by BamHI digest. A total of 28 vORFs were synthesized for Y2H_GFP_ and 27 for Y2H_HIS3_: NSP1-16 (except NSP11), S, E, M, N and ORFs 3A, 3B, 3D, 6, 7A, 7B, 8, 9B, 9C and 10^62–64^ (Supplementary Table 1).

Y2H_HIS3_ vORF entry clones were verified by full-length Sanger sequencing. As NSP10 had a one-base deletion, it was excluded from further experiments. vORFs were moved to the destination vectors pPC86 (N-terminal AD fusion, CEN origin)^3,65^ and pHiDEST-DB (N-terminal DB fusion, CEN origin)^4^ by Gateway cloning and confirmed by PCR. For Y2H_GFP_, barcoded ‘prey’ (pAR068: C-terminal AD fusion, 2μ origin/pHiDEST-AD: N-terminal AD fusion, CEN origin), and ‘bait’ (pHiDEST-DB: N-terminal DB fusion, CEN origin) destination vectors were generated using published protocols^4^, with the integration of the barcode locus at the SacI restriction site as described^26^. Single barcoded plasmid containing colonies were picked, arrayed into 384-well plates with 80 μl LB agar supplemented with 100 μg ml^−1^ carbenicillin and 35 μg ml^−1^ chloramphenicol (LB + Carb+CM) per well and incubated at 37°C for 16 h. Barcode sequences were identified using a modified Kiloseq procedure^66^ using an Illumina NextSeq 500 and analyzed as previously described^4,26,66^. Y2H_GFP_ vORFs and human ACE2 were moved by Gateway cloning into barcoded destination plasmids^4,26^ pHiDEST-AD (N-terminal AD fusion, CEN origin (low copy number)) and pHiDEST-DB (N-terminal DB fusion, CEN origin (low copy number)) such that each ORF was linked to two to six barcodes in every configuration. Gateway cloning was performed individually and for ORF–barcode pairs using Sanger sequencing (TCAG, The Hospital for Sick Children) (Supplementary Table 13).

### Generation of HuSCI_HIS3_

The Y2H_HIS3_ screening pipeline is essentially as previously described^65^. AD-Y and DB-X vORFs were transformed into yeast strains Y8800 (MATa) and Y8930 (MATα), respectively. NSP1 autoactivated as DB fusion and not screened in this orientation. DB-X vORFs were individually mated with 99 pools of ~188 AD-tagged human ORFs each, from human ORFeome v9.1 comprising 17,472 ORFs^26,67^ (hORFeome9.1). For the reverse orientation, yeast with 27 AD-Y vORFs were pooled and mated against DB-X hORFeome9.1. Primary screening in both configurations was performed twice to increase sampling sensitivity. Unless otherwise noted, all yeast incubations are at 30°C, overnight without shaking.

For primary screening, saturated haploid AD-Y and DB-X yeast cultures were spotted on top of each other on yeast extract peptone dextrose (YEPD) agar (1%) plates and incubated for 24 h. Yeast were replica plated onto selective synthetic complete media lacking leucine, tryptophan and histidine (SC-Leu-Trp-His) + 1 mM 3-AT (3-amino-1,2,4-triazole)^3,65^ (3-AT plates) and incubated for 72 h. From growing spots up to three colonies were picked and cultured in SC-Leu-Trp liquid medium for 2 d. For second phenotyping, cultures were spotted on diploid selection plates, incubated for 2 d and replica plated on 3-AT-plates and SC-Leu-His + 1 mM 3-AT + 1 mg per liter cycloheximide plates to identify spontaneous DB-X autoactivators^2^. Positive scoring colonies (growth on 3-AT-plates, no growth on cycloheximide plates) were picked, and ORFs were identified by Sanger sequencing^65^. For threefold verification, yeast strains corresponding to the identified human interaction partners were picked from archival glycerol stocks, cultured in liquid medium and mated (as described above) one-by-one against all vORFs, processed as described above and then scored. Colony growth was scored using a custom dilated convolutional neural network^68^. For training, previous datasets of more than 1,500 images of biochemically and functionally validated binary Y2H studies were used^3^. Each image was scaled to achieve equal pixel distance between the yeast spots of different images. The images were cropped and sliced, and the mean grayscale image of all spots on a plate was calculated. With this dataset, a simple front-end prediction module was trained consisting of six dilated convolutional layers with exponential increasing dilation rate and two dense layers at the end. After each layer except the last, a Leaky-ReLU activation was added^69^. The model was optimized with a combination of Softmax and Cross entropy and an Adam Optimizer^70^. The model achieved an accuracy >0.9 during all folds of a tenfold cross-fold validation. All positive scores were confirmed by a trained researcher. The verification step was done in triplicate and protein pairs scoring positive in at least two repeats were considered bona fide Y2H interactors. One representative colony of all interaction pairs was picked from selective plates to confirm the identities of X and Y by Sanger sequencing^65^.

### Generation of HuSCI_GFP_

#### Barcoded ORFeomes

The barcoded human ORFeome consisting of 16,747 fully sequence-verified human ORFs with ~95% ORFs represented by two unique barcodes was previously described^26^. The barcoded bait and prey collections were arranged into a 10-by-10 screening matrix consisting of 10 DB and 10 AD groups, each containing ~1,400 ORFs with two distinct sets of unique barcodes, and ~200 ORFs with a single unique barcode set. Barcoded SARS-CoV-2 plasmids were transformed individually into RY3011 (AD plasmids) and RY3031 (DB plasmids) (genotypes in Supplementary Table 14). Transformed colonies were copied on fresh plates, incubated, scraped off and pooled to make glycerol stocks of all the barcoded SARS-CoV-2 ORFs plus the human ORF ACE2 in each plasmid configuration (with two or more barcodes per ORF).

#### Mating of pooled haploid yeast

Multiple pooled matings were performed using the frozen haploid pools. Each of the 10 human ORF pools (in C-terminal AD fusion plasmids with 2μ origin; pAR068) were separately mixed with the pool of SARS-CoV-2 ORFs plus human ACE2 (in N-terminal DB fusion plasmids with CEN origins; pHiDEST-DB). A separate mating was done between the SARS-CoV-2 pools in both AD and DB fusion, CEN origin plasmids (pHiDEST-AD, pHiDEST-DB). Negative controls were included in each mating and all matings were calculated to achieve >100× coverage of possible barcode combinations considering viability and mating efficiency. Procedurally, equal amounts of each haploid strain were mixed, the mixture was spread on 2x YEPD plus adenine agar plates (YPAD) and incubated for 24 h. Colonies on each mating plate were collected and re-spread across 20 15 cm SC-Leu-Trp plates supplemented with histidine (8 mM) and incubated for 72 h. These plates were then scraped off to make assay-ready pooled diploid glycerol stocks for each of the 11 groups.

#### Selection of yeast with interacting pair of DB-X and AD-Y by FACS

Pool of glycerol stocks were inoculated into 1-liter flasks with a starting vCFU of 30 M and incubated at 200 rpm for 24 h. Negative controls were started as 10 ml cultures and processed in parallel. ‘Presort’ cultures were prepared for each sample (2 × 10 ml cultures with OD_600_ 10) with doxycycline added (10 μg ml^−1^) to these cultures to induce barcode swapping while these cultures were incubated for 24 h^4^. To prepare for fluorescence-activated cell sorting (FACS), cells were concentrated by centrifugation (500 × *g*, 5 min) and resuspended in PBS to a final OD_600_ of 10. Propidium iodide (4 mg liter^−1^) was added to identify dead yeast cells during FACS. Using the diploid negative control, the FACS gate for GFP-positive cells was set to capture 0.1% of GFP-negative cells, yielding a 0.01% false positive rate. Then, 100 million cells per group were sorted, and GFP-positive cells for each sample were plated on 10 SC-Leu-Trp+Ade+10x His (8 mM) plates and incubated for 72 h. Colonies were collected by scraping, centrifuged and resuspended into 2 × 10 ml cultures (OD_600_ = 10). Doxycycline (10 μg ml^−1^) was added to induce barcode swapping, and cultures were incubated for 24 h, when plasmid DNA was extracted. Fused barcodes were PCR amplified with primers that attach modified Illumina i5 and i7 adapters to uniquely identify each sample. Following agarose gel analysis of PCR products, the bright band at ~350 bp was purified using a NucleoSpin Gel and PCR Clean-up kit. DNA concentrations were measured for each sample using a Qubit (Invitrogen, Q32851) and, guided by DNA concentration, samples were pooled to ensure equal sequencing depth relative to the number of protein pairs tested. After primer-dimer removal, DNA was quantified by qPCR, and the pooled NGS library was sequenced on an Illumina NextSeq using a mid- or high-output 150-cycles kit.

#### Read counting based on expected barcodes

The sequencing data were demultiplexed using bcl2fastq2 (v2.20.0.422) provided by Illumina with the following command: ‘bcl2fastq -r 10 -p 20 -w 10 –no-lane-splitting –barcode-mismatches 1 –adapter-stringency 0.7 –ignore-missing-bcls –ignore-missing-filter –ignore-missing-positions’. After demultiplexing, the fastq files were aligned to the group specific reference files using bowtie2^71^ with the following parameters:

For read 1: -q –norc –local –very-sensitive-local -t -p 23 –reorder.

For read 2: -q –nofw –local –very-sensitive-local -t -p 23 –reorder.

Reference files contained expected barcode sequences for the ORFs in each group. After alignments, reads with mapping quality scores <20 were removed. Following successful BFG barcode recombination^4^, paired-end reads map to up-up or dn-dn when an interaction is present. The number of reads mapping to up-up and dn-dn were counted separately and merged as the final read count. The pipeline was implemented in Python v2.7.

#### Interaction scoring

For virus–host interactions, we used the product of marginal frequencies of bait and prey strains^4^ to estimate the abundance of each diploid bait–prey strain in the presort condition (‘PreSort’). The interaction score was defined by

$$\begin{array}{lll} {IS_{ij}} &=& {\frac{{f_{ij}^{\,GFP}}}{{f_{ij}^{\Pr eSort}}}}\\ {f_{ij}^{\, \Pr eSort}} &=& \mathop {\sum}\limits_i {c_{ij}^{\Pr eSort}/\mathop {\sum}\limits_j {\left[ {\mathop {\sum}\limits_i {c_{ij}^{\Pr eSort}} } \right]}}\\f_j^{\, \Pr eSort} &=& \mathop {\sum}\limits_j {c_{ij}^{\Pr eSort}} /\mathop {\sum}\limits_i {\left[ {\mathop {\sum}\limits_j {c_{ij}^{\Pr eSort}} } \right]}\\ {f_j^{\, \Pr eSort}} &=& \max \left( {f_i^{\, \Pr eSort},f_{AD}^{\, Floor}} \right) \times \max \left( {f_j^{\, \Pr eSort},f_{DB}^{\, Floor}} \right) \\f_{AD}^{\, Floor} &=& 10^{ - 5}f_{DB}^{\, Floor} = 10^{ - 4}\\ {f_{ij}^{\, GFP}} &=& {c_{ij}^{GFP}/\mathop {\sum}\limits_{ij} {c_{ij}^{GFP}} } \end{array}$$

with the following variables: *c*, read count; *i*, AD barcode count; *j*, DB barcode count; *f*, frequency.

For every DB barcode, we used the 960 AD null barcodes to define the thresholds leading to a 1% false positive rate. An interaction was accepted as positive only if the ORF pair interaction score was above this threshold for two or more barcode pairs. For intraviral screening, we accepted as interactions those protein pairs for which the frequency of barcode pairs was 1,000 times greater than the median frequency of the corresponding DB barcode for three or more independent barcode pairs, similar to the scoring method previously used for BFG-Y2H with HIS3-based growth selection^4^.

#### Pairwise retesting

Candidate interaction pairs for HuSCI_GFP_ were verified in a pairwise HIS3 growth-based Y2H assay as described above (Y2H_HIS3_ verification step), with minor modifications. Barcode replicates of candidate human AD-Y and viral DB-X were pooled prior to mating. vORFs NSP1 and NSP12 were omitted from this retesting due to DB autoactivation. After mating, colonies were replica plated on SC-Leu-Trp-His and 3AT-plates. After 72–96 h of yeast growth, these pairwise tests were scored according to the standardized scoring method used for the Y2H_HIS3_ screen^3,65^. Interaction pairs scoring ≥3 were considered bona fide Y2H interactions.

### Estimating completeness using the interactome framework

Assay sensitivity (S_a_) is defined as the fraction of true interactions that can be detected by a given assay. Sampling sensitivity (S_s_) is defined as a fraction of detectable true interactions that can be recovered by the pipeline used. Overall sensitivity of a given screen S can be calculated as S = S_a_ × S_s_. In pairwise settings S_s_ = 1 and the assay sensitivity is given by the fraction of hsPRS-v1/v2 pairs that score positive. Y2H_HIS3_ was benchmarked previously^5^ and has an assay sensitivity of S_a-HIS3_ = 21.7%. Sampling sensitivity of Y2H_HIS3_ after two repeats in two orientations has been shown to be S_s-HIS3_ = ~60%^65^, yielding a screening sensitivity of S_HIS3_ = S_a-HIS3_ × S_s-HIS3_ = 0.217 × 0.6 = 13%. Given that Y2H_HIS3_ screen had a search space completeness of 83% (T_HIS3_ = 83%), the overall completion of HuSCI_HIS3_ is C_HIS3_ = T_HIS3_ × S_HIS3_ = 0.83 × 0.13 = 10.8%.

A different version of Y2H_GFP_ using low-copy plasmids and N-terminally fused hybrid proteins (lcnY2H_GFP_) was benchmarked using 84 pairs of hsPRS-v1 and 92 pairs of hsRRS-v1. Flow cytometry was used to score interactions based on percentage of singlets in GFP-positive gate, which was set using empty bait and prey constructs. In addition, lcnY2H_GFP_ was benchmarked in a pooled setting using all possible combinations of proteins constituting 78 hsPRS-v2 and 77 hsRRS-v2 pairs supplemented with a set of 14 pairs of Y2H-positive controls defined as calibration set^4^. The experiment was carried out and interactions were scored as described above, except that no empirical null distribution was used. lcnY2H_GFP_ recovered 12 out of 82 (S_a-lcnGFP_ = 15%) hsPRS-v1 pairs when tested in a pairwise single bait–prey configuration and 8 of 92 (9%, S_s-lcnGFP_ = 9/15 = 60%) hsPRS-v2 + calibration set pairs when tested in a pooled single bait–prey configuration, yielding S_lcnGFP_ = S_a-lcnGFP_ × S_s-lcnGFP_ = 0.15 × 0.6 = 9%. It has been previously shown that using high-copy C-terminal fusions increases sensitivity by ~33% without affecting precision^26^. Thus, screening sensitivity of Y2H_GFP_ was modeled from that of lcnY2H_GFP_ as S_GFP_ = S_lcnGFP_ × 1.33 = 9% × 1.33 = 12%. Given that Y2H_GFP_ covered 70% (T_GFP_ = 70%) of all possible virus–human protein combinations, the completion level of the Y2H_GFP_ dataset is C_GFP_ = T_GFP_ × S_GFP_ = 0.70 × 0.12 = 8.4%. Only 4 out of 28 (14.2%) hsPRS-v1 pairs detected by the union of Y2H_HIS3_ and lcnY2H_GFP_ were detected with both methods, indicating a high degree of orthogonality (that is, different detection profiles of the methods used). In addition, Y2H_GFP_ implemented in this study includes further differences such as high-copy and C-terminal fusion constructs for human proteins. Therefore, we conservatively estimate 90% orthogonality between Y2H_HIS3_ and Y2H_GFP_ (that is, ~90% of detected interactions are different: O_HIS3+GFP_ = 90%). Thus, we estimate that the fraction of all true interactions captured by our merged interactome maps is C_HIS3+GFP_ = (C_HIS3_ + C_GFP_) × O_HIS3+GFP_ ≅ (0.108 + 0.084) × 0.9 = 17.3%. Given the uncertainties associated with derivation of screening sensitivity, we estimate lower and higher bounds to be 15% (S_GFP_ = 9%, excluding inferred gain in sensitivity due to high-copy C-terminal fusions) and 22% (S_GFP_ = 13.5%, S_s-HIS3_ = 70% and O_HIS3+GFP_ = 100%), respectively.

### Pairwise Y2H testing of previously identified SARS-CoV-1 interactions

We identified 97 unique curated binary interactions with SARS-CoV-1 and human interaction partners^8^ (Supplementary Table 2). For 77 of these, reagents to test interactions with SARS-CoV-2 orthologues were available in the barcoded human ORFeome. These involved 63 human proteins, 60 of which were covered by two barcode sets and three by a single barcode set. These were tested according to the ‘pairwise retesting’ protocol (above). Successful interactions were indicated by colony growth of both replicates in either condition.

### Pairwise Y2H testing with SARS-CoV-2 variants

Lineage-defining mutations for the SARS-CoV-2 ‘variants of concern’ as defined by the Centers for Disease Control and Prevention (Alpha, Beta, Gamma and Delta) were obtained from CoV-Spectrum^72,73^ and mapped to the SARS-CoV-2 reference genome (NCBI accession number NC_045512.2). To generate variant ORFs, Y2H_HIS3_ plasmids were used as template for mutation PCR (primers in Supplementary Table 12). Mutation PCR reaction products were transformed and sequence verified. Plasmids containing the desired mutation were directly transformed into yeast and processed in pairwise mating as described above. A complete list of mutations generated is shown in Supplementary Table 12. SARS-CoV-2 proteins for which interactions were identified in AD-fusions (N and E) were tested only against the identified interactors. All other variant proteins were tested against all HuSCI interactors. In total, 19 individual mutations in 14 unique variant proteins from 9 different viral proteins were tested. Four proteins with 8 cloned variants had interactors in HuSCI_HIS3_, 1 protein with a single cloned variant had interactors in HuSCI_GFP_ and 4 proteins with 5 variants had no HuSCI interactors.

### yN2H validation

Using Gateway cloning, ORFs from the indicated subsets (Supplementary Table 3) were transferred into pDEST-N2H plasmids (pDEST-N2H-N1, -N2, -C1, and -C2) containing a *LEU2* (N1/C1 vectors) or a *TRP1* (N2/C2 vectors) auxotrophy marker and transformed into haploid *Saccharomyces cerevisiae* Y8800 (MATa) and Y8930 (MATα) strains. For cross-plate calibration, two protein pairs from the hsPRS-v2, with different N2H signal intensities, were included in duplicate on every plate (NCBP1/NCBP2 and SKP1/SKP2). Virus–human protein pairs were randomly distributed across the plates and tested together with hsPRS-v2/hsRRS-v2, which were in separate plates.

Overnight-grown haploid cultures were mated by mixing 5 μl of each haploid strain in 160 μl YEPD medium followed by overnight incubation. To measure background, all interactor ORFs were also mated with yeast with empty F1 or F2 plasmids. After mating, 10 μl culture each was inoculated into 160 μl SC-Leu-Trp and grown overnight, and then 50 μl was reinoculated into 1.2 ml SC-Leu-Trp and incubated for 24 h while shaking at 900 rpm. Cells were harvested (6,000 x *g*, 15 min), and the supernatant was discarded. Each yeast cell pellet was fully resuspended in 100 μl NanoLuc Assay solution^6^. Homogenized solutions were transferred into white flat-bottom 96-well plates and incubated in the dark (for 1 h at room temperature). Luminescence was evaluated for each sample with 2 s integration time. To score X–Y protein pairs, a normalized luminescence ratio (NLR) was calculated corresponding to the raw luminescence value of the tested pair (X-Y) divided by the maximum luminescence value from one of the two controls (X-Fragment 2 or Fragment 1-Y)^6^. The 1% RRS threshold was based on the vhRRS and determined using the R quantile function.

### Enrichment of previously known, phospho-regulated or RNA-binding host targets

From IntAct^8^ (version: April 28, 2020), 2,151 human proteins reported to have binary interactions with any virus protein were defined as ‘previously known host targets’. 2,005 of these ORFs were interrogated by our experiment, and further considered. HuSCI contained 61 previously known host targets. 2,254 human proteins that change phosphorylation changes upon SARS-CoV-2 infection were identified from A549 and Vero E6 cell lines^9,10^, of which 2,007 were interrogated by our experiment and 37 are in HuSCI. 139 experimentally identified human proteins specifically bound to SARS-CoV-2 RNA (vRICs) and 335 human proteins with altered RNA-binding activity upon SARS-CoV-2 infection (cRICs) were obtained from a recent RNA-interactome study^11^. Then, 121 vRICs and 294 cRICs were interrogated by our experiment; 5 HuSCI proteins were vRICs, and 13 HuSCI proteins were cRICs. All the observations were tested for enrichment using Fisher’s exact tests and by permutation tests with 10,000 permutations.

### GO enrichment analysis

gProfiler^74^ (database versions: Ensembl 104, Ensembl Genomes 51 and Wormbase ParaSite 15) was applied to identify enriched functional categories in HuSCI, AP-MS^9,12–15^ and BioID studies^16–18^. The hORFeome9.1, which was used for contactome mapping, served as the background for HuSCI, otherwise the universal annotated human genes. ‘Inferred from electronic annotations’ annotations were excluded. Adjusted *P* values were calculated using the Benjamini–Hochberg procedure. Functional terms with a hypergeometric *P* < 0.05 and term size between 5 and 1,000 were collected and enrichment calculated as the ratio between observed and expected gene counts. To categorize HuSCI host proteins, five meta categories inspired by the functional enrichment analysis results were used, namely ‘immune response’ (GO:0006955), ‘viral process’ (GO:0016032), ‘protein ubiquitination’ (GO:0016567), ‘cytoskeleton’ (GO:0005856) and ‘vesicle-mediated transport’ (GO:0016192). Human proteins related to these categories were obtained from the AmiGO 2 (ref. ^75^) (July 2021), and HuSCI host proteins were categorized based on their annotation to these meta categories.

### Domain enrichment of host interacting proteins

Structural domains in human targets were identified from Pfam release 34.0 (ref. ^76^) (March 2021). Interactions of viral proteins with human interactors that have common domains were defined as shared-domain interactions and counted for HuSCI. The procedure was repeated for 1,000 randomized HuSCI networks (degree-preserved random rewiring). The significance of every viral protein–human domain was assessed by Fisher’s exact tests (Supplementary Table 6) using the number of V-D, V-!D, !V-D, and !V-D interacting pairs, in which V and D correspond to the viral protein and human domain of interest, and !V and !D to the rest of viral proteins and domains in the HuSCI network, respectively. We identified as enriched associations those with at least two V-D interactions and *P* < 0.05. We repeated the process for 1,000 randomized HuSCI networks (see above). Multiple domain copies in a given human protein were counted once.

### NF-κB reporter assays

HEK293 (RRID: CVCL_0045, DSMZ) were cultured in complete DMEM (high glucose) supplemented with 10% fetal calf serum, 100 U ml^−1^ penicillin and 100 µg ml^−1^ streptomycin and maintained in humidified atmosphere at 5% CO_2_ at 37°C. For the reporter assay, 1 × 10^6^ HEK293 cells were seeded in a 60-mm cell culture dish one day before transfection. Transfection was done using the calcium phosphate protocol using 10 ng NF-κB reporter plasmid (6 × NF-κB firefly luciferase pGL2), 50 ng pTK reporter (Renilla luciferase) and expression vectors (Flag-IKKb (pRK5), Flag-A20 (pEF4) and SARS-CoV-2 constructs (pMH)) using a total of up to 6 μg DNA. Briefly, the DNA was diluted in 200 µl 250 mM CaCl_2_ solution (Carl Roth, 5239.1), vortexed and added dropwise to 200 µl 2 × HBS (50 mM HEPES (pH 7.0), 280 mM NaCl, 1.5 mM Na_2_HPO_4_ × 2 H_2_O, pH 6.93) while gently vortexing. After 15-min incubation at room temperature, the mix was added dropwise to cell culture dishes. Transfection media was replaced after 6-h incubation with complete DMEM. Then, 24 h after transfection cells were stimulated with 20 ng ml−1 TNF-α for 4 hours. Luciferase activity was measured using the dual luciferase reporter kit (Promega, E1980) according to the manufacturer’s protocol. The firefly and Renilla luminescence was determined with a luminometer (Berthold Centro LB960 microplate reader, software MikroWin 2010) and quantified in relative light units (RLU). NF-κB induction was specified as the ratio of firefly luminescence (RLU) to Renilla luminescence (RLU). Significance of relative NF-κB transcriptional activity was assessed via one-way ANOVA with Dunnett’s multiple comparisons. Data evaluation was performed in GraphPad Prism v7.04.

Protein expression was verified by western blot of lysates. Briefly, proteins were separated by SDS-PAGE and transferred on polyvinylidene fluoride membranes. Membranes were blocked with 5% milk in 1 × PBS + 0.1 % Tween-20 (PBS-T) for 1 h at room temperature. Primary antibodies in 2.5% milk in PBS-T were incubated overnight at 4°C, the membranes were washed three times with PBS-T and secondary antibodies were incubated (1.25% milk/PBS-T) for 1 h at room temperature. Anti-actin beta (SCBT, sc-47778), anti-FLAG M2 (Sigma-Aldrich, F3165) and anti-HA (Sigma-Aldrich, 11583816001, RRID:AB_514505) were used at a 1:1,000 dilution. Secondary antibody (Jackson ImmunoResearch, Jim-715-035-150) was used at a 1:10,000 dilution. For detection of horseradish peroxidase-catalyzed enhanced chemiluminescence, LumiGlo reagent (CST, 7003S) was used.

For generation of *IKBKG* KO HEK293 cells, oligonucleotides coding sgRNAs targeting exon 3 (5′-TGCATTTCCAAGCCAGCCAG-3′) or exon 2 (5′- GCTGCACCATCTCACACAGT-3′) were cloned into px458 (Addgene, 48138). HEK293 were transfected with 5 µg plasmid by standard calcium phosphate transfection. After one day, GFP-positive cells were sorted with a MoFlo cell sorter (Beckman Coulter, Cytomation) and seeded in 96-well plates at dilutions of 0.5–5.0 cells per well. Single-cell clones were expanded and screened for loss of IKBKG expression by western blot (RRID: AB_2124846). IKBKG-negative clones were verified by amplifying and sequencing a region of genomic DNA encompassing the sites targeted by PCR (exon 3: forward primer 5′-CTGGCCAACACGTACTTTTA-3′, reverse primer 5′-GGTTACGGTGAGCGAAGGCTC-3′; exon 2: forward primer 5′- CTGACATCTCCCTCCACAAAC-3′ and reverse primer 5′-GGAGCTGGAATGAACCTTCC-3′).

### Functional effects on viral replication

#### Selection of host-target candidates

To evaluate if identified host targets are involved in viral replication, the following HuSCI proteins involved in host immune regulation^77^ and viral life cycle regulation^51,78–80^ by enriched GO terms in this study were selected: G3BP1, G3BP2, TRAF2, USP25, EIF2AK2, REL, IKBKG and KLC1.

#### Engineering of hACE2-expressing cells

A549 cells were seeded at 5 × 10^5^ cells per well in six-well cell culture plates and cultured in DMEM with 10% FCS and 1% penicillin/streptomycin at 37°C and 5% CO_2_ (standard media). After 24 h culture medium was replaced by fresh medium containing 4.5 × 10^7^ transduction units hACE2 lentivirus per well and incubated for 4 hours at 37°C and 5% CO_2_. The lentiviral inoculum was then replaced with 2 ml DMEM 10% FCS and 1% penicillin/streptomycin. After 24 h, the transduction was repeated with the same steps as above. Cell surface expression of hACE2 was monitored by FACS using the AttuneNxT Flow Cytometer (Thermo Fisher Scientific) and results were analyzed with FlowJo v10 Software (BD Life Sciences). The resulting cells are referred to as A549-hACE2.

#### Generation of KO cell lines

KO cells were generated using the target-specific CRISPR-Cas9-HDR (homology-directed recombination) KO directed technology developed by Santa Cruz Biotechnology, which enables selection of KO cells with puromycin and red fluorescent protein (Supplementary Table 15). Briefly, A549-hACE2 cells were seeded at 2.5 × 10^6^ cells in T25 flasks and standard media. After 24 h, cells were cotransfected with 7.5 µg each of KO and HDR plasmids for the previously described targets and 15 µg KO plasmid for the mock KO, from Santa Cruz Biotechnology using FuGene (Promega, E2312). After 72 h, KO cells were selected with 2 µg/ml puromycin (Invivogen, ant-pr-1) for 3 d, and mock KO cells were treated with the same volume of Hepes solution (Sigma-Aldrich, 51558). One week later, red fluorescent protein-positive cells were sorted by flow cytometry. DNA from 2 × 10^6^ cells was extracted and region of interest was amplified for each KO, except KLC1, in a 25-µl PCR using 50 ng genomic DNA and using one primer in the genomic DNA and one primer in the insert (primers are listed in Supplementary Table 15). KLC1 KO was verified by amplifying the sg-directed Cas9 region that had no corresponding HDR with one primer on each side of the region; the PCR product was purified using Nucleospin Gel and PCR Clean-up (Machery-Nagel, 11992242) and KO confirmed by Sanger sequencing.

#### Assessment of SARS-CoV-2 infection in A549-hACE2 KO versus wild-type cells

Wild-type and KO A549-hACE2 cells were seeded at 1 × 10^6^ cells per well in 12-well plates and standard media. After 24 h, cells were infected at a multiplicity of infection (MOI) of 10^−3^, with SARS-CoV-2 isolate hCoV19/France/GE1973/2020 (*n* = 3, biological replicates). Total RNA was extracted from infected cells at 72 h after infection, and SARS-CoV-2 replication was assessed by RT-qPCR using Orf1ab primers (5′-ATGAGCTTAGTCCTGTTG-3′; 3′-CTCCCTTTGTTGTGTTGT-5′) (*n* = 9, three technical replicates per biological replicate). GAPDH was used for normalization. Viral RNA was quantified according to the ∆∆Ct standard method^81^. The effect of gene KO on viral replication was determined using the wild-type ORF1ab RNA level as a control as shown in the following equation: 2^−(∆∆Ct)^ = 2^−(∆Ct KO − ∆Ct WT)^. Significance of the KO effect was calculated against the mock KO using an ordinary one-way nonparametric ANOVA Kruskal-Wallis with Dunn’s multiple comparisons test using GraphPad Prism v9.

#### Assessment of the viability of the KO cell lines

A total of 8.0 × 10^5^ cells of each KO cell line were seeded in a white 96-well plate and incubated at 37°C and 5% CO_2_ for 24 h. Cell media was replaced with DMEM and incubated at 37 °C and 5% CO_2_ for 72 h. Cell viability was measured using Cell Titer-Glo Luminescent Cell Viability Assay kit (Promega, G7750). Luminescence was measured on a Centro XS luminometer (Berthold; integration time, 0.5 s). Wild-type cells served as the reference and significance of cell viability was calculated against the mock KO using an ordinary one-way nonparametric ANOVA Kruskal–Wallis with Dunn’s multiple comparisons test using GraphPad Prism v9.

### Genes ranked by number of publications

Publication counts are derived from the gene2pubmed file from NCBI, downloaded on 16 November 2021. Only protein-coding genes were considered. For visualization, but not statistical assessment, of genes with equal numbers of publications, order was determined by random shuffling. *P* values were calculated by Mann–Whitney *U* test, with Bonferroni correction. Black dots indicate the mean; error bars represent the 95% confidence interval generated from 1,000 bootstrap samples.

### Tissue specificity analysis

The Tissue Atlas dataset was obtained from the HPA database^21^ (version 2021.04.09). The HPA categories ‘tissue enriched’, ‘group enriched’ and ‘tissue enhanced’ were combined with ‘tissue-specific’, ‘low tissue specificity’ was denoted as ‘common’ and the ‘not detected’ category was not included in this analysis. A total of 11,069 of 19,670 genes (56.3%) in the HPA dataset were defined as tissue specific, and 8,385 of 19,670 genes (42.6%) showed common expression profiles. Tissue distribution differences were determined using Fisher’s exact test with Bonferroni correction.

SARS-CoV-2 organotropism data were obtained from post mortem examinations^22,82^. The RNA tissue-specific NX value (normalized transcripts per million) was extracted and used to denote whether the gene is specifically expressed in a given tissue. Tissues from the Tissue Atlas were combined into organ systems and used to assess host-target tissues. Significance was evaluated by Fisher’s exact test with Bonferroni correction.

### Identification of genetic variation in host targets and network communities

Host network communities were identified using the OCG hierarchical community clustering algorithm on the Human Reference Interactome^26,83^ as implemented in the linkcomm R package (V1.0-13) using ‘centered cliques’ as initial class system^84^. A total of 3,603 communities with a minimum size of 4 were found, of which 204 contained a significant number of virus interactors (that is, were significantly targeted) (nominal *P* < 0.05, Fisher’s exact test; Supplementary Table 8). A community was annotated to a function if a GO term was enriched (FDR < 0.05) or if ≥20% or ≥30% of the annotated constituent proteins shared an annotation^85^ (Supplementary Table 8). From AP-MS-based association studies^9,12–15^, 57, 43, 18 and 17 significantly targeted communities were found, respectively (nominal *P* < 0.05, Fisher’s exact test; Supplementary Table 8).

Uniformly processed GWAS summary statistics were downloaded for 114 traits from the GTEx GWAS analysis^41,86^. MAGMA^87^ analysis was implemented in R 3.6.1 and consists of three steps: first, GWAS summary statistics across all single-nucleotide polymorphisms (SNPs) within a gene region are aggregated into a gene-level association *P* value. Next, the gene-level *P* value is transformed to a z-score (using the inverse normal cumulative distribution function). Finally, z-scores across all genes are modeled as a function of gene set membership and the default gene-level covariates (gene size in number of SNPs, the gene density (a measure of within-gene linkage disequilibrium), the inverse mean minor allele count) using a linear model. Association between gene set membership and GWAS z-scores is tested based on the null hypothesis beta = 0 for the coefficient associated with the gene set membership indicator variable. All targets, and the targeted network communities, were considered gene sets. Entrez gene IDs were used on the human genome assembly 38. Individual MAGMA analyses were performed for each trait based on summary statistics and linkage disequilibrium structure from the 1,000 genomes European reference panel always conditioning on default gene-level covariates (for example, gene length). For each gene set, standard error normalized beta coefficients constituted the association score, with larger values indicating greater chance of getting significant association. Following Benjamini–Hochberg multiple hypothesis correction, gene set–trait associations with FDR < 0.05 were selected. These pairs were subjected to follow-up analysis. SNPs localizing within genes of enriched gene sets were selected, and genes containing SNPs with GWAS *P* < 5.0 × 10^−8^ were selected for the enriched traits, which were considered ‘GWAS hits’. As control the analysis was repeated for the 3,399 network communities that were not significantly targeted (Supplementary Table 8). For both targeted and non-targeted communities the probability of observing traits that are linked to COVID-19 outcomes was assessed. A literature survey identified 35 traits clinically linked to COVID-19 (score 2 in Supplementary Table 8), 18 ‘related to immune function’ and 61 without connection. For the enrichment analysis we focused on the ‘COVID-linked’ traits; traits ‘related to immune function’ are also indicated in Fig. 3. Finally, Fisher’s exact test was used to assess the significance traits being linked to COVID-19 (score 2) vs not (scores 0 and 1) in traits that are associated with not-virus- targeted communities (*P* = 0.5) vs virally targeted communities (*P* = 0.01). For the control analysis of AP-MS targeted communities, only genetic variation related to COVID-19 severity was evaluated. The contactome-targeted communities with significant GWAS trait associations were numbered 1–31.

### Small-molecule inhibition

Remdesivir (Bio-Techne, 7226/10) and USP25/28 inhibitor AZ1 (Bio-Connect, HY-117370-5mg) were dissolved in DMSO. HEK293-ACE2 and Vero E6 (3 × 10^4^ cells per well) were plated in white 96-well plates. After 24 h, cells were infected with SARS-CoV-2 (ref. ^54^) (0.01 MOI) containing a nanoluciferase reporter and treated with the compounds in a 12-point twofold dilution series with 0–10 µM concentration. Each condition was done in triplicate, except for AZ1, which was done in quadruplicate for HEK293-ACE2 and one replicate for Vero E6. Cells were cultured for 24 h, and luminescence was quantified^88^. Cell viability was measured using the Cell Titer-Glo Luminescent Cell Viability Assay kit (Promega, G7750). EC_50_ values were calculated via the variable slope model in GraphPad Prism v9.

### Reporting summary

Further information on research design is available in the Nature Research Reporting Summary linked to this article.

## Online content

Any methods, additional references, Nature Research reporting summaries, source data, extended data, supplementary information, acknowledgements, peer review information; details of author contributions and competing interests; and statements of data and code availability are available at 10.1038/s41587-022-01475-z.

## Supplementary information

Supplementary Information Legends to Extended Data Figs. 1–5 and description of content of Supplementary Tables 1–15.

Reporting Summary

Supplementary Table 1 List of interactions identified in this study. **a**, Total list of viral proteins used in both Y2H screens. Annotations are given for the putative function of individual proteins, as well as amino acid sequences for each Y2H screen. **b**, Total list of interactions between viral and host proteins in HuSCI. The screen in which the interaction was found (HuSCI_HIS3_ and/or HuSCI_GFP_) is specified, as well as interactions also found by the four AP-MS studies and the three BioID studies. Host proteins found by any of the other association studies via different viral proteins are also indicated. **c**, Total list of PPIs among viral proteins (IntraSCI) identified by Y2H_GFP_. The overlap with a previous intraviral PPI study is also indicated^7^.

Supplementary Table 2 Supplementary Table 2. A curated list of previously identified binary interactions between SARS-CoV-1 and human proteins and identified orthologous SARS-CoV-2-human pairs (HuSCI_ORTH_). The information provided includes the publication in which the interaction was reported. The columns ‘autoactivator’, ‘no growth’ and ‘no human clone’ indicate whether the interaction was examined.

Supplementary Table 3 Orthogonal N2H assay validation of HuSCI and IntraSCI along with positive (hsPRS-v2 and vhLit-BM) and negative (hsRRS-v2 and vhRRS) benchmarking sets. **a**, List of PPIs in virus–host literature binary multiple reference set (vhLit-BM). The number of methods by which the interaction was identified is indicated. **b**, List of protein pairs in virus–host Random Reference Set (vhRRS). **c**, Luminescence values for orthogonal N2H validation of HuSCI (HuSCI_HIS3_ and HuSCI_GFP_) and IntraSCI, as well as positive (hsPRS-v2 and vhLit-BM) and negative (hsRRS-v2 and vhRRS) benchmarking sets. **d**, Number of hits above threshold (1%vhRRS) and total number of pair configurations tested. **e**, *P* value (two-tailed hypergeometric test) calculated for all possible network pairs of validation.

Supplementary Table 4 Tissue specificity and organotropism. **a**, Tissue specificity and organotropism across SARS-CoV-2 infected tissues of HPA, HuSCI, SARS-CoV-2 co-complex and BioID datasets (Gordon et al.^14,15^, Stukalov et al.^10^, Li et al.^16^, Nabeel-Shah et al.^17^, Laurent et al.^18^, St-Germain et al.^19^ and Samavarchi-Tehrani et al.^20^). **b**, Summary statistics for tissue specificity of datasets in panel a, relative to HPA (Extended Data Fig. 2b). **c**, Organotropism analysis across SARS-CoV-2 infected tissues of datasets in panel a. The percentage of genes within a certain dataset with specific organotropism (‘tissue-specific’ expression in tissues grouped into organ systems) is shown (Extended Data Fig. 2c). **d**, Summary statistics of organotropism analysis for datasets in panel a, relative to HPA.

Supplementary Table 5 Functions enriched in HuSCI, four AP-MS and three BioID based networks. **a**, HuSCI. **b**, Gordon et al.^14,15^. **c**, Stukalov et al.^10^. **d**, Li et al.^16^. **e**, Nabeel-Shah et al.^17^. **f**, Laurent et al.^18^. **g**, St-Germain et al.^19^. **h**, Samavarchi-Tehrani et al.^18^.

Supplementary Table 6 Analysis of shared domain associations in HuSCI. **a**, Statistical analysis of HuSCI shared domain associations. **b**, HuSCI domain associations of all PPIs.

Supplementary Table 7 Functional enrichment analysis of the HuSCI proteins linking viral to critical illness proteins from subnetwork of proteins in COVID-19 ‘critical illness’-associated loci and their direct interactors in HuRI. Subnetwork proteins that are viral targets in HuSCI are listed in Supplementary Table 8h.

Supplementary Table 8 GWAS trait associations in significantly targeted HuRI communities by HuSCI and subnetwork of GWAS candidate protein-coding genes and their first neighbors. **a**, Protein membership in significantly targeted HuRI communities by HuSCI. **b**, Statistical enrichment of HuSCI host targets in HuRI communities. **c**, Functional enrichment of significantly targeted HuRI communities by HuSCI. **d**, GO terms of significantly targeted HuRI communities by majority rule of protein members at 30% threshold. **e**, GO terms of significantly targeted HuRI communities by majority rule of protein members at 20% threshold. **f**, GWAS traits associated with significantly targeted communities by HuSCI. **g**, GWAS traits associated with non-targeted and not significantly targeted communities. **h**, Metadata for COVID-19 associations of all queried GWAS traits. **i**, Subnetwork of proteins in COVID-19 critical-illness-associated loci and their direct interactors in HuRI. **j**, Association of subnetwork proteins in COVID-19 critical-illness-associated loci and their direct interactors in HuRI.

Supplementary Table 9 Quantification of NF-κB reporter activity by individual viral proteins in HEK293 cells. **a**, NF-κB and TK (control) transcriptional reporter activity in the absence and presence of individual viral proteins with and without TNF-α stimulation. **b**, Summary statistics for data from panel a (Extended Data Fig. 4a). **c**, NF-κB and TK (control) transcriptional reporter activity at different amounts of transfected viral protein-encoded plasmid without TNF-α stimulation. **d**, NF-κB and TK (control) transcriptional reporter activity at different amounts of transfected viral protein-encoded plasmid with TNF-α stimulation. **e**, Summary statistics for data from panels c and d (Extended Data Fig. 4b). **f**, NF-kB transcriptional reporter activity in wild-type and different IKBKG KO cell lines with TNF-α stimulation or transfected with viral NSP14 protein-encoded plasmid. **g**, Summary statistics for data from panel f (Extended Data Fig. 4c).

Supplementary Table 10 Quantification of viral replication in A549-ACE2 cells in the presence and absence of CRISPR-Cas9-mediated KOs of selected host interactors. **a**, Raw Ct values (qPCR) for viral replication in A549-ACE2 cells. **b**, Fold change (ORF1ab / GAPDH) relative to wild-type (WT) cells. **c**, Summary statistics for viral replication assay (Extended Data Fig. 4d). **d**, Raw luminescence values (Cell Titer Glo) for the KO cell viability assay. **e**, Analysis of KO cell viability data. **f**, Summary statistics for cell viability assay.

Supplementary Table 11 Quantification of viral replication in HEK293 and Vero-E6 cells treated with AZ1 or Remdesivir. **a**, Raw (RLU), as well as relative (%) luminescence values of viral replication in HEK293 cells treated with AZ1. **b**, Raw (RLU), as well as relative (%) luminescence values of cell viability for AZ1 treated HEK293 cells. **c**, Summary analysis of viral replication in HEK293 cells treated with AZ1 (EC_50_). **d**, Raw (RLU), as well as relative (%) luminescence values of viral replication in Vero E6 cells treated with AZ1. **e**, Raw (RLU), as well as relative (%) luminescence values of cell viability for AZ1 treated Vero E6 cells. **f**, Summary analysis of viral replication in Vero E6 cells treated with AZ1 (EC50). **g**, Raw (RLU), as well as relative (%) luminescence values of viral replication in HEK293 cells treated with Remdesivir. **h**, Raw (RLU), as well as relative (%) luminescence values of cell viability for Remdesivir treated HEK293 cells. **i**, Summary analysis of viral replication in HEK293 cells treated with Remdesivir (EC_50_). **j**, Raw (RLU), as well as relative (%) luminescence values of viral replication in Vero E6 cells treated with Remdesivir. **k**, Raw (RLU), as well as relative (%) luminescence values of cell viability for Remdesivir treated Vero E6 cells. **m**, Summary analysis of viral replication in Vero E6 cells treated with Remdesivir (EC_5_0)

Supplementary Table 12 Materials used for construction of variant clones. **a**, Mutations of SARS-CoV-2 variant proteins. **b**, Primers for gene point mutations.

Supplementary Table 13 Total list of barcoded viral clones used for Y2H_GFP_. **a**, Total list of barcoded viral clones in AD-Nterm-Cen Y2H destination vector for Y2H_GFP_. **b**, Total list of barcoded viral clones in DB-Nterm-Cen Y2H destination vector for Y2H_GFP_.

Supplementary Table 14 Genotypes of toolkit strains used in Y2H_GFP_.

Supplementary Table 15 CRISPR-Cas9 and HDR plasmids used to make A549-ACE2 KO cell lines and primer sequences for verification of KO for viral replication assay.

## Data Availability

The protein-protein interaction (PPI) data from this publication have been submitted to the IMEx (http://www.imexconsortium.org) consortium through IntAct and assigned the identifier IM-28880 (ref. ^89^). All data from the study are included in the article and associated files. Source data are provided with this paper. The following data were obtained from the respective original publications: phosphorylation changes upon SARS-CoV-2 infection^9,10^; RNA-binding changes upon SARS-CoV-2 infection^11^; AP-MS virus–host association data: Gordon et al.^12,13^, Stukalov et al.^9^, Li et al.^14^, Nabeel-Shah et al.^15^; BioID virus–host proximity data: Laurent et al.^16^, St-Germain et al.^17^ Samavarchi-Tehrani et al.^18^; human expression data: Human Proteome Atlas^21^, SARS-CoV-2 organotropism^22,82^; human host interactome: HuRI^26^; GWAS data for severe COVID-19 illness^32,33^; and GWAS summary statistics for 114 traits: doi:10.5281/ZENODO.3518299. Interaction data for other viruses were downloaded from IntAct^8^ (version: 28 April 2020). Publication counts were downloaded from gene2pubmed (NCBI) on 16 November 2021. Source data are provided with this paper.

## Source data

Source Data Fig. 4 Source Data Fig. 4c: unprocessed western blot.
Source Data Extended Data Fig. 4 Source Data Extended Data Fig. 4d,e: unprocessed western blot.

## Code availability

All source code related to this paper is available via GitHub (https://github.com/INET-HMGU/SARS-CoV-2-contactome)^90^.

## References

[1] Nalbandian A (2021). Post-acute COVID-19 syndrome. Nat. Med..

[2] Yu H (2008). High-quality binary protein interaction map of the yeast interactome network. Science.

[3] Altmann M (2020). Extensive signal integration by the phytohormone protein network. Nature.

[4] Yachie N (2016). Pooled-matrix protein interaction screens using Barcode Fusion Genetics. Mol. Syst. Biol..

[5] Braun P (2009). An experimentally derived confidence score for binary protein-protein interactions. Nat. Methods.

[6] Choi SG (2019). Maximizing binary interactome mapping with a minimal number of assays. Nat. Commun..

[7] Li Y (2021). SARS-CoV-2 induces double-stranded RNA-mediated innate immune responses in respiratory epithelial-derived cells and cardiomyocytes. Proc. Natl. Acad. Sci. U. S. A..

[8] Orchard S (2014). The MIntAct project–IntAct as a common curation platform for 11 molecular interaction databases. Nucleic Acids Res.

[9] Stukalov A (2021). Multilevel proteomics reveals host perturbations by SARS-CoV-2 and SARS-CoV. Nature.

[10] Bouhaddou M (2020). The global phosphorylation landscape of SARS-CoV-2 infection. Cell.

[11] Kamel W (2021). Global analysis of protein-RNA interactions in SARS-CoV-2 infected cells reveals key regulators of infection. Mol. Cell.

[12] Gordon DE (2020). Comparative host-coronavirus protein interaction networks reveal pan-viral disease mechanisms. Science.

[13] Gordon DE (2020). A SARS-CoV-2 protein interaction map reveals targets for drug repurposing. Nature.

[14] Li J (2021). Virus-host interactome and proteomic survey reveal potential virulence factors influencing SARS-CoV-2 pathogenesis. Med (N Y).

[15] Nabeel-Shah S (2022). SARS-CoV-2 nucleocapsid protein binds host mRNAs and attenuates stress granules to impair host stress response. iScience.

[16] Laurent, E. M. N. et al. Global BioID-based SARS-CoV-2 proteins proximal interactome unveils novel ties between viral polypeptides and host factors involved in multiple COVID19-associated mechanisms. Preprint at *bioRxiv*10.1101/2020.08.28.272955 (2020).

[17] St-Germain, J. R. et al. A SARS-CoV-2 BioID-based virus-host membrane protein interactome and virus peptide compendium: new proteomics resources for COVID-19 research. Preprint at *bioRxiv*10.1101/2020.08.28.269175 (2020).

[18] Samavarchi-Tehrani, P. et al. A SARS-CoV-2–host proximity interactome. Preprint at *bioRxiv*10.1101/2020.09.03.282103 (2020).

[19] Wierbowski SD (2021). A 3D structural SARS-CoV-2–human interactome to explore genetic and drug perturbations. Nat. Methods.

[20] Callard F, Perego E (2021). How and why patients made long covid. Soc. Sci. Med..

[21] Uhlén M (2015). Proteomics. Tissue-based map of the human proteome. Science.

[22] Dorward DA (2021). Tissue-specific immunopathology in fatal COVID-19. Am. J. Respir. Crit. Care Med..

[23] Zhao X (2020). LY6E restricts entry of human coronaviruses, including currently pandemic SARS-CoV-2. J. Virol..

[24] Garcia-Moreno M (2019). System-wide profiling of RNA-binding proteins uncovers key regulators of virus infection. Mol. Cell.

[25] Zanzoni A, Spinelli L, Ribeiro DM, Tartaglia GG, Brun C (2019). Post-transcriptional regulatory patterns revealed by protein-RNA interactions. Sci. Rep..

[26] Luck K (2020). A reference map of the human binary protein interactome. Nature.

[27] Kruse T (2021). Large scale discovery of coronavirus-host factor protein interaction motifs reveals SARS-CoV-2 specific mechanisms and vulnerabilities. Nat. Commun..

[28] Ferrari S (2001). Mutations of CD40 gene cause an autosomal recessive form of immunodeficiency with hyper IgM. Proc. Natl. Acad. Sci. U. S. A..

[29] de Vries L, Gat-Yablonski G, Dror N, Singer A, Phillip M (2014). A novel MKRN3 missense mutation causing familial precocious puberty. Hum. Reprod..

[30] Zhong Q (2016). An inter-species protein-protein interaction network across vast evolutionary distance. Mol. Syst. Biol..

[31] Sahni N (2015). Widespread macromolecular interaction perturbations in human genetic disorders. Cell.

[32] Pairo-Castineira E (2021). Genetic mechanisms of critical illness in COVID-19. Nature.

[33] COVID-19 Host Genetics Initiative. (2021). Mapping the human genetic architecture of COVID-19. Nature.

[34] Whyte P (1988). Association between an oncogene and an anti-oncogene: the adenovirus E1A proteins bind to the retinoblastoma gene product. Nature.

[35] Weßling R (2014). Convergent targeting of a common host protein-network by pathogen effectors from three kingdoms of life. Cell Host Microbe.

[36] Ostaszewski M (2021). COVID19 Disease Map, a computational knowledge repository of virus-host interaction mechanisms. Mol. Syst. Biol..

[37] Soveg FW (2021). Endomembrane targeting of human OAS1 p46 augments antiviral activity. eLife.

[38] Cifuentes-Muñoz N, Dutch RE, Cattaneo R (2018). Direct cell-to-cell transmission of respiratory viruses: the fast lanes. PLoS Pathog.

[39] Zhu Y (2021). A genome-wide CRISPR screen identifies host factors that regulate SARS-CoV-2 entry. Nat. Commun..

[40] Daniloski Z (2021). Identification of required host factors for SARS-CoV-2 infection in human cells. Cell.

[41] Barbeira AN (2021). Exploiting the GTEx resources to decipher the mechanisms at GWAS loci. Genome Biol.

[42] Bliddal S (2021). Acute and persistent symptoms in non-hospitalized PCR-confirmed COVID-19 patients. Sci. Rep..

[43] Whiting A, Reyes JVM, Ahmad S, Lieber J (2021). Post-COVID-19 fatigue: a case of infectious hypothyroidism. Cureus.

[44] Mohan M, Perry BI, Saravanan P, Singh SP (2021). COVID-19 in people with schizophrenia: potential mechanisms linking schizophrenia to poor prognosis. Front. Psychiatry.

[45] VanderWeele TJ (2010). Genetic self knowledge and the future of epidemiologic confounding. Am. J. Hum. Genet..

[46] Li, T. et al. SARS-CoV-2 Nsp14 activates NF-κB signaling and induces IL-8 upregulation. Preprint at *bioRxiv*10.1101/2021.05.26.445787 (2021).

[47] Hadjadj J (2020). Impaired type I interferon activity and inflammatory responses in severe COVID-19 patients. Science.

[48] Sun G (2021). Comparative transcriptomic analysis of SARS-CoV-2 infected cell model systems reveals differential innate immune responses. Sci. Rep..

[49] Costela-Ruiz VJ, Illescas-Montes R, Puerta-Puerta JM, Ruiz C, Melguizo-Rodríguez L (2020). SARS-CoV-2 infection: the role of cytokines in COVID-19 disease. Cytokine Growth Factor Rev.

[50] Hayden MS, Ghosh S (2014). Regulation of NF-κB by TNF family cytokines. Semin. Immunol..

[51] Lin D (2015). Induction of USP25 by viral infection promotes innate antiviral responses by mediating the stabilization of TRAF3 and TRAF6. Proc. Natl. Acad. Sci. U. S. A..

[52] Wrigley JD (2017). Identification and characterization of dual inhibitors of the USP25/28 deubiquitinating enzyme subfamily. ACS Chem. Biol..

[53] Xie X (2020). An infectious cDNA clone of SARS-CoV-2. Cell Host Microbe.

[54] Hou YJ (2020). SARS-CoV-2 reverse genetics reveals a variable infection gradient in the respiratory tract. Cell.

[55] Grodzki M (2022). Genome-scale CRISPR screens identify host factors that promote human coronavirus infection. Genome Med.

[56] Chang L-J, Chen T-H (2021). NSP16 2’-O-MTase in coronavirus pathogenesis: Possible prevention and treatments strategies. Viruses.

[57] Alshiraihi IM, Klein GL, Brown MA (2021). Targeting NSP16 methyltransferase for the broad-spectrum clinical management of coronaviruses: managing the next pandemic. Diseases.

[58] Li Q (2020). The impact of mutations in SARS-CoV-2 spike on viral infectivity and antigenicity. Cell.

[59] Syed AM (2021). Rapid assessment of SARS-CoV-2 evolved variants using virus-like particles. Science.

[60] Kim D-K (2020). A comprehensive, flexible collection of SARS-CoV-2 coding regions. G3.

[61] Wu F (2020). A new coronavirus associated with human respiratory disease in China. Nature.

[62] Wu A (2020). Genome composition and divergence of the novel coronavirus (2019-nCoV) originating in China. Cell Host Microbe.

[63] Jungreis I (2021). Conflicting and ambiguous names of overlapping ORFs in the SARS-CoV-2 genome: A homology-based resolution. Virology.

[64] Gibson DG (2009). Enzymatic assembly of DNA molecules up to several hundred kilobases. Nat. Methods.

[65] Altmann M, Altmann S, Falter C, Falter-Braun P (2018). High-quality yeast-2-hybrid interaction network mapping. Curr. Protoc. Plant Biol..

[66] Weile J (2017). A framework for exhaustively mapping functional missense variants. Mol. Syst. Biol..

[67] The ORFeome Collaboration. (2016). The ORFeome collaboration: a genome-scale human ORF-clone resource. Nat. Methods.

[68] Fisher, Y. & Koltun, V. Multi-Scale Context Aggregation by Dilated Convolutions. CoRR abs/1511.07122 (JMLR.org, 2016): n. pag.

[69] Maas, A. L, Hannun, A. Y & Ng, A. Y. Rectifier nonlinearities improve neural network acoustic models. *Proceedings of the 30th International Conference on Machine Learning*, **30** (Atlanta, GA, 2013).

[70] Kingma, D. P. & Ba, J. Adam: a method for stochastic optimization. Preprint at *arXiv* https://arxiv.org/abs/1412.6980 (2014).

[71] Langmead B, Salzberg SL (2012). Fast gapped-read alignment with Bowtie 2. Nat. Methods.

[72] Chen C (2021). CoV-Spectrum: analysis of globally shared SARS-CoV-2 data to identify and characterize new variants.. Bioinformatics.

[73] Shannon P (2003). Cytoscape: a software environment for integrated models of biomolecular interaction networks. Genome Res.

[74] Reimand J, Kull M, Peterson H, Hansen J, Vilo J (2007). g:Profiler—a web-based toolset for functional profiling of gene lists from large-scale experiments. Nucleic Acids Res.

[75] Carbon S (2009). AmiGO: online access to ontology and annotation data. Bioinformatics.

[76] Mistry J (2021). Pfam: the protein families database in 2021. Nucleic Acids Res.

[77] Shin C (2017). MKRN2 is a novel ubiquitin E3 ligase for the p65 subunit of NF-κB and negatively regulates inflammatory responses. Sci. Rep..

[78] Götte B (2019). Separate domains of G3BP promote efficient clustering of alphavirus replication complexes and recruitment of the translation initiation machinery. PLoS Pathog..

[79] Hosmillo M (2019). Noroviruses subvert the core stress granule component G3BP1 to promote viral VPg-dependent translation. eLife.

[80] Liu S, Dominska-Ngowe M, Dykxhoorn DM (2014). Target silencing of components of the conserved oligomeric Golgi complex impairs HIV-1 replication. Virus Res..

[81] Livak KJ, Schmittgen TD (2001). Analysis of relative gene expression data using real-time quantitative PCR and the 2(-Delta Delta C(T)) method. Methods.

[82] Meinhardt J (2020). Olfactory transmucosal SARS-CoV-2 invasion as a port of central nervous system entry in individuals with COVID-19. Nat. Neurosci..

[83] Becker E, Robisson B, Chapple CE, Guénoche A, Brun C (2012). Multifunctional proteins revealed by overlapping clustering in protein interaction network. Bioinformatics.

[84] Kalinka AT, Tomancak P (2011). linkcomm: an R package for the generation, visualization, and analysis of link communities in networks of arbitrary size and type. Bioinformatics.

[85] Chapple CE (2015). Extreme multifunctional proteins identified from a human protein interaction network. Nat. Commun..

[86] Barbeira, A. N. *et al*. GWAS and GTEx QTL integration. *Zenodo*10.5281/ZENODO.3518299 (2019).

[87] de Leeuw CA, Mooij JM, Heskes T, Posthuma D (2015). MAGMA: generalized gene-set analysis of GWAS data. PLoS Comput. Biol..

[88] Coutant EP (2020). Bioluminescence profiling of NanoKAZ/NanoLuc luciferase using a chemical library of coelenterazine analogues. Chemistry.

[89] Kim, D.K. et al. IM-28880. IMEx. https://www.ebi.ac.uk/legacy-intact/query/pubid:unassigned2933;jsessionid=E9D9D501AAC618B88078DBD0BD47AEFA?conversationContext=1 (2022).

[90] Kim, D.K. et al. SARS-CoV-2-contactome. GitHub. https://github.com/INET-HMGU/SARS-CoV-2-contactome (2022).

[91] Barron E (2020). Associations of type 1 and type 2 diabetes with COVID-19-related mortality in England: a whole-population study. Lancet Diabetes Endocrinol.

[92] Leong A (2021). Cardiometabolic risk factors for COVID-19 susceptibility and severity: a Mendelian randomization analysis. PLoS Med..

[93] Nikniaz Z, Somi MH, Dinevari MF, Taghizadieh A, Mokhtari L (2021). Diabesity associates with poor COVID-19 outcomes among hospitalized patients. J. Obes. Metab. Syndr..

[94] Aung N, Khanji MY, Munroe PB, Petersen SE (2020). Causal inference for genetic obesity, cardiometabolic profile and COVID-19 susceptibility: a Mendelian randomization study. Front. Genet..

[95] Freuer D, Linseisen J, Meisinger C (2021). Impact of body composition on COVID-19 susceptibility and severity: a two-sample multivariable Mendelian randomization study. Metabolism.

[96] Wang C (2020). Red cell distribution width (RDW): a prognostic indicator of severe COVID-19. Ann. Transl. Med..

[97] Ouyang S-M (2020). Temporal changes in laboratory markers of survivors and non-survivors of adult inpatients with COVID-19. BMC Infect. Dis..

[98] Kearns SM (2021). Reduced adiponectin levels in patients with COVID-19 acute respiratory failure: a case-control study. Physiol Rep..

[99] Hypothyroidism is associated with prolonged COVID-19-induced anosmia: a case-control study. *J. Neurol. Neurosurg. Psychiatry* **20**, jnnp–2021–326587 (2021). 10.1136/jnnp-2021-326587 33879534

[100] Brancatella A (2020). Subacute thyroiditis after SARS-CoV-2 infection. J. Clin. Endocrinol. Metab..

[101] Nemani K (2021). Association of psychiatric disorders with mortality among patients with COVID-19. JAMA Psychiatry.

[102] Zhu Z (2020). Association of obesity and its genetic predisposition with the risk of severe COVID-19: analysis of population-based cohort data. Metabolism.

[103] Derikx LAAP (2021). Clinical outcomes of COVID-19 in patients with inflammatory bowel disease: a nationwide cohort study. J. Crohns. Colitis.

[104] Dar HY, Azam Z, Anupam R, Mondal RK, Srivastava RK (2018). Osteoimmunology: the between bone and immune system. Front. Biosci..

