# Supplementary material for: A proteome-scale map of the SARS-CoV-2–human contactome
Source: Nat Biotechnol. 2022 Oct 10;41(1):140–9. doi: 10.1038/s41587-022-01475-z (PMC9849141; doi:10.1038/s41587-022-01475-z)
Supplement: Supplementary file 1 — Legends to Extended Data Figs. 1–5 and description of content of Supplementary Tables 1–15. [file 41587_2022_1475_MOESM1_ESM.pdf]

nature biotechnology

Article

https://doi.org/10.1038/s41587-022-01475-z

A proteome-scale map of the SARS-CoV-2–human contactome

In the format provided by the authors and unedited

SUPPLEMENTARY INFORMATION GUIDE

Extended Data Fig. 1 | Screening space, orthogonal validation and comparison of HuSCI and IntraSCI to previous SARS-CoV-2 related datasets. a, Schematic of the experimental contactome mapping pipelines (left) and screening space for each of the two parallel $Y2H_{HIS}$ and $Y2H_{GFP}$ screens (right). Proportional overlap is given relative to the union of protein pairs tested by both methods. **b**, Rate at which interactions are detected by yN2H for HuSCI and IntraSCI, as well as positive (hsPRS-v2 and vhLit-BM) and negative (hsRRS-v2 and vhRRS) benchmark sets, across stringency thresholds, error band: standard error of proportion. **c**, Left: overlap of previously identified intra-viral SARS-CoV-2 interactions and IntraSCI; right: actual overlap (arrow) compared to n = 10,000 randomized control networks. One-sided, empirical *P* = 0.0046. **d**, Left: overlap of host targets identified in HuSCI and differentially phosphorylated proteins following infection by SARS-CoV-2; right: actual overlap (arrow) compared to n = 10,000 randomized control networks. One-sided, empirical *P* < 0.0001. **e**, Left: overlap of host targets identified in HuSCI and RNA Binding Proteins (RBPs) demonstrating differential RNA binding upon SARS-CoV-2 infection; right: actual overlap (arrow) compared to n = 10,000 randomized control networks. One-sided, empirical *P* = 0.022.

Extended Data Fig. 2 | Comparison of HuSCI with SARS-CoV-2 association and proximity datasets. a, Overlap of viral-human protein pairs between HuSCI, four AP-MS and three BioID based datasets (Gordon et al.[14,15], Stukalov et al.[10], Li et al.[13], Nabeel-Shah et al.[16], Laurent et al.[17], St-Germain et al.[19], Samavarchi-Tehrani et al.[18]). **b**, Statistical analysis of representation of host targets in common and specific expression groups from datasets in (**a**), compared to the Human Protein Atlas22 (HPA) (Fisher's exact test with Bonferroni correction). **c**, Organotropism analysis across SARS-CoV-2 infected organs from datasets in (**b**). The percentage of genes within each dataset with specific organotropism ('tissue specific' expression in tissues grouped into organ systems). **b** and **c**, Full analysis is shown in **Supplementary Table 4. d**, Proportion of HuSCI host interactors per SARS-CoV-2 protein in which the human protein has: domains present in other interactors of the viral protein (shared); domains not present in other interactors of the viral protein (unique); no structural domains. Full analysis is shown in **Supplementary Table 6**.

Extended Data Fig. 3 | Traits associated with COVID-19 severity. a, Table showing COVID-19 critical illness associated loci from two GWAS meta-analyses[33,34]. Locus-associated proteins present in HuRI are marked in bold. **b**, Genes in indicated COVID-19 datasets ranked across the human genome by number of publications. Error bars are 95% confidence intervals of the mean, calculated by 1,000 bootstrap samples (from top to bottom n = 45, 170, 383, 876, 29, 75, 25, 71, 233, 46, 49,

45, 15, 33, 10, 71, 97, 47, 58, 9, 46, 22, 20, 23, 39). **c**, Virus-interactor enrichment in contactome: number of direct SARS-CoV-2 protein interacting HuSCI proteins in a HuRI subnetwork formed by proteins encoded by COVID-19 critical illness associated loci[34] (marked in bold in table (**a**)) and their first level interactors (arrow) compared to n = 10,000 randomized control networks (grey distribution). One-sided, empirical *P* = 0.012. **d**, Virus-interactor enrichment in co-complex associations: number of SARS-CoV-2 associated human proteins in two AP-MS based studies[10,15] in a subnetwork formed by proteins encoded by COVID-19 critical illness associated loci[33,34] (marked in bold in table in (**a**)) and their first level interactors (arrow) either in HuRI or BioPlex 3.0. The comparisons are against n = 10,000 randomized control networks (grey distribution). One-sided, empirical *P* values are shown for each dataset. **e**, Upset plots showing number of communities targeted by SARS-CoV-2 (left) and associated with severe COVID-19 (right) in HuSCI and AP-MS based datasets. **f**, Table showing 15 traits for genetic variation identified within targeted network communities. An association with severe COVID-19 comorbidities is indicated, as well as trait references: T2D_UKBS[63–65], BMIA[64,66], FAT_UKBS[67], HRET[68], RET[68], HC_UKBS[66,69], ADPN[70], HYPOTHY_UKBS[44,71,72], SCZ_UKBS[45,73], GIANT_HIP[64,74], IBD_UKBS[75], OST_UKBS[76], EGG_PHF, GIANT_HEIGHT, NEUROT_UKB. **g**, Grouping of 31 network communities with significantly associated traits shown in **Fig. 3d** by protein membership measured by Jaccard similarity according to legend.

Extended Data Fig. 4 | Effect of viral proteins on NF-κB reporter activity and of viral interactors on viral replication. **a**, Tables showing statistical details of NF-κB transcriptional reporter activity in the absence and presence of selected viral proteins under unstimulated (top) and TNFα stimulated (bottom) conditions. One-way ANOVA with Dunnett’s multiple comparisons test, n = 3, adjusted *P* values are shown. **b**, Table showing statistical details of NF-κB transcriptional reporter activity at different amounts of transfected viral protein-encoded plasmid under unstimulated (left) and TNFα stimulated conditions (right). One-way ANOVA with Dunnett’s multiple comparisons test, n = 3 and n = 6, respectively, adjusted *P* values are shown. **a** and **b**, Raw data and full analysis is shown in **Supplementary Table 9**. **c**, Table showing statistical details of NF-κB transcriptional reporter activity under unstimulated (left), TNFα-stimulated (middle) and NSP14-induced conditions in WT and IKBKG-KO HEK293 cells (two-way ANOVA with Dunnett’s multiple comparisons test, n = 3), adjusted *P* values are shown. **d**, Representative anti-IKBKG (top) western blot demonstrating levels of IKBKG in WT and three independent IKBKG knock-out clones of HEK293 cells relative to actin beta (ACTB) loading controls (bottom). **e**, Representative anti-hemagglutinin (HA) western blot demonstrating levels of tagged NSP14 protein in NF-κB induction experiments relative to actin beta (ACTB) loading controls (bottom). **f**, Table showing statistical details of viral replication in wild-type, mock KO and

CRISPR KOs of the indicated HuSCI host proteins. Kruskal-Wallis with Dunn's multiple comparisons test, n = 9. Adjusted *P* values are shown. **g**, Cell viability of mock KO and CRISPR KOs of the indicated HuSCI host proteins relative to WT cells. Kruskal-Wallis with Dunn's multiple comparisons test, n = 3. Adjusted, exact *P* values are shown. **f** and **g**, Raw data, exact *P* values, and full analysis is shown in **Supplementary Table 10**. **h**, Cell viability and relative replication of icSARS-CoV-2-nanoluciferase in HEK293 cells (left) and Vero E6 cells (right) at different concentrations of remdesivir. The EC50 values shown for each cell line were calculated with a variable slope model. Error bars: standard deviation of the mean, n = 3 biological repeats, full analysis in **Supplementary Table 11**.

Extended Data Fig. 5 | Mutations of SARS-CoV-2 variants affect specific interactions with uSCI host targets. a, $Y2H_{HIS3}$ yeast growth on selective plates of HuSCI interaction partners as DB-fusion proteins tested against AD-fusion of the SARS-CoV-2 Nucleocapsid (AD-N) protein (Wuhan-Hu1, original screen) and AD-N containing 'lineage defining' amino acid substitutions: D3L and S235F (α-strain), T205I (β-strain), or P80R (γ-strain). Shown is one representative result of 5 repeats. **b**, $Y2H_{HIS3}$ yeast growth on selective plates of HuSCI interaction partners as DB-fusion proteins tested against AD-fusion of the SARS-CoV-2 Envelope (AD-E) protein (Wuhan-Hu1, original screen) or AD-E containing 'lineage defining' substitution P71L (β-strain). Shown is one representative $Y2H_{HIS3}$ result on selective media, out of 2 repeats. **a** - **c**, Black circles indicate changes in yeast colony growth between human proteins tested against viral variant ORFs or the originally screened Wuhan strain ORFs observed consistently across all repeats. **c**, AD-empty control plate for **a, b** indicates lack of autoactivation. **d**, Layout of DB-fusion HuSCI interactors (purple) tested with AD fusion SARS-CoV-2 proteins or AD-empty control, respectively in **a - c**. N/A indicates human interactors not part of this study. *Yeast spots in column 6 failed to grow for technical reasons.

Supplementary Table 1. List of interactions identified in this study. a, Total list of viral proteins used in both Y2H screens. Annotations are given for the putative function of individual proteins, as well as amino acid sequences for each Y2H screen. **b,** Total list of interactions between viral and host proteins in HuSCI. The screen in which the interaction was found ($HuSCI_{HIS3}$ and/or $HuSCI_{GFP}$) is specified, as well as interactions also found by the four AP-MS studies and the three BioID studies. Host proteins found by any of the other association studies via different viral proteins are also indicated. **c,** Total list of PPIs among viral proteins (IntraSCI) identified by $Y2H_{GFP}$. The overlap with

a previous intra-viral PPI study is also indicated[8].

Supplementary Table 2. A curated list of previously identified binary interactions between SARS-CoV-1 and human proteins and identified orthologous SARS-CoV-2-human pairs (HuSCI$_{\mathbf{ORTH}}$). The information provided includes the publication in which the interaction was reported. The columns “autoactivator”, “no growth” and “no human clone” indicate whether the interaction was examined.

Supplementary Table 3. Orthogonal N2H assay validation of HuSCI and IntraSCI along with positive (hsPRS-v2 and vhLit-BM) and negative (hsRRS-v2 and vhRRS) benchmarking sets. a, List of PPIs in virus-host literature binary multiple reference set (vhLit-BM). The number of methods by which the interaction was identified is indicated. **b,** List of protein pairs in virus-host Random Reference Set (vhRRS). **c,** Luminescence values for orthogonal N2H validation of HuSCI (HuSCI$_{HIS3}$ and HuSCI$_{GFP}$) and IntraSCI, as well as positive (hsPRS-v2 and vhLit-BM) and negative (hsRRS-v2 and vhRRS) benchmarking sets. **d,** Number of hits above threshold (1%vhRRS) and total number of pair-configurations tested. **e,** *P*-value (two-tailed hypergeometric test) calculated for all possible network pairs of validation.

Supplementary Table 4. Tissue specificity and organotropism. a, Tissue specificity and organotropism across SARS-CoV-2 infected tissues of HPA, HuSCI, SARS-CoV-2 co-complex and BioID datasets. (Gordon et al.[14,15], Stukalov et al.[10], Li et al.[13], Nabeel-Shah et al.[16], Laurent et al.[17], St-Germain et al.[19], Samavarchi-Tehrani et al.[18]). **b,** Summary statistics for tissue specificity of datasets in (**a**), relative to HPA (**Extended Data Fig. 2b**). **c,** Organotropism analysis across SARS-CoV-2 infected tissues of datasets in (**a**). The percentage of genes within a certain dataset with specific organotropism (‘tissue specific’ expression in tissues grouped into organ systems) is shown (Extended Data Figure 2c). **d,** Summary statistics of organotropism analysis for datasets in (**a**), relative to HPA.

Supplementary Table 5. Functions enriched in HuSCI, four AP-MS and three BioID based networks. a, HuSCI **b,** Gordon et al.[14,15] **c,** Stukalov et al.[10] **d,** Li et al.[13] **e,** Nabeel-Shah et al.[16] **f,** Laurent et al.[17] **g,** St-Germain et al.[19] **h,** Samavarchi-Tehrani et al.[18].

Supplementary Table 6. Analysis of shared domain associations in HuSCI. a, Statistical analysis of HuSCI shared domain associations. **b,** HuSCI domain associations of all PPIs.

Supplementary Table 7. Functional enrichment analysis of the HuSCI proteins linking viral to critical illness proteins from subnetwork of proteins in COVID-19 'critical illness'-associated loci and their direct interactors in HuRI. Subnetwork proteins that are viral targets in HuSCI are listed in Supplementary Table 8h.

Supplementary Table 8. GWAS trait associations in significantly targeted HuRI communities by HuSCI and subnetwork of GWAS candidate protein-coding genes and their first neighbors. a, Protein membership in significantly targeted HuRI communities by HuSCI. **b,** Statistical enrichment of HuSCI host targets in HuRI communities. **c,** Functional enrichment of significantly targeted HuRI communities by HuSCI. **d,** Gene ontology terms of significantly targeted HuRI communities by majority rule of protein members at 30% threshold. **e,** Gene ontology terms of significantly targeted HuRI communities by majority rule of protein members at 20% threshold. **f**, GWAS traits associated with significantly targeted communities by HuSCI. **g**, GWAS traits associated with non-targeted and not significantly targeted communities. **h**, Metadata for COVID-19 associations of all queried GWAS traits. **i,** Subnetwork of proteins in COVID-19 'critical illness'-associated loci and their direct interactors in HuRI. **j,** Association of subnetwork proteins in COVID-19 'critical illness'-associated loci and their direct interactors in HuRI.

Supplementary Table 9. Quantification of NF-κB reporter activity by individual viral proteins in HEK293 cells. a, NF-κB and TK (control) transcriptional reporter activity in the absence and presence of individual viral proteins with and without TNFα stimulation. **b,** Summary statistics for data from (**a**) (Extended Data Fig. 4a). **c,** NF-κB and TK (control) transcriptional reporter activity at different amounts of transfected viral protein-encoded plasmid without TNFα stimulation. **d,** NF-κB and TK (control) transcriptional reporter activity at different amounts of transfected viral protein-encoded plasmid with TNFα stimulation. **e**, Summary statistics for data from (**c**) and (**d**) (Extended Data Fig. 4b). **f**, NF-kB transcriptional reporter activity in wild type and different IKBKG KO cell lines with TNFα stimulation or transfected with viral NSP14 protein-encoded plasmid. **g**, Summary statistics for data from (**f**) (Extended Data Fig. 4c).

Supplementary Table 10. Quantification of viral replication in A549-ACE2 cells in the presence and absence of CRISPR/Cas9-mediated knockouts (KO) of selected host interactors. a, Raw Ct values (qPCR) for viral replication in A549-ACE2 cells. **b,** Fold change (ORF1ab / GAPDH) relative to wild type (WT) cells. **c,** Summary statistics for viral replication assay (Extended Data Fig. 4d). **d**,

Raw luminescence values (Cell-titer Glo) for the KO cell viability assay. **e**, Analysis of KO cell viability data. **f**, Summary statistics for cell viability assay.

Supplementary Table 11. Quantification of viral replication in HEK293 and Vero-E6 cells treated with AZ1 or Remdesivir. a, Raw (RLU), as well as relative (%) luminescence values of viral replication in HEK293 cells treated with AZ1. **b**, Raw (RLU), as well as relative (%) luminescence values of cell viability for AZ1 treated HEK293 cells. **c**, Summary analysis of viral replication in HEK293 cells treated with AZ1 (EC50). **d**, Raw (RLU), as well as relative (%) luminescence values of viral replication in VeroE6 cells treated with AZ1. **e**, Raw (RLU), as well as relative (%) luminescence values of cell viability for AZ1 treated VeroE6 cells. **f**, Summary analysis of viral replication in VeroE6 cells treated with AZ1 (EC50). **g**, Raw (RLU), as well as relative (%) luminescence values of viral replication in HEK293 cells treated with Remdesivir. **h**, Raw (RLU), as well as relative (%) luminescence values of cell viability for Remdesivir treated HEK293 cells. **i**, Summary analysis of viral replication in HEK293 cells treated with Remdesivir (EC50). **j**, Raw (RLU), as well as relative (%) luminescence values of viral replication in VeroE6 cells treated with Remdesivir. **k**, Raw (RLU), as well as relative (%) luminescence values of cell viability for Remdesivir treated VeroE6 cells. **m**, Summary analysis of viral replication in VeroE6 cells treated with Remdesivir (EC50).

Supplementary Table 12. Materials used for construction of variant clones. a, Mutations of SARS-CoV-2 variant proteins. **b,** Primers for gene point mutations.

Supplementary Table 13. Total list of barcoded viral clones used for $Y2H_{GFP}$. a, Total list of barcoded viral clones in AD-Nterm-Cen Y2H destination vector for $Y2H_{GFP}$. **b,** Total list of barcoded viral clones in DB-Nterm-Cen Y2H destination vector for $Y2H_{GFP}$.

Supplementary Table 14. Genotypes of toolkit strains used in $Y2H_{GFP}$.

Supplementary Table 15. CRISPR Cas9 and HDR plasmids used to make A549-ACE2 KO cell lines and primer sequences for verification of KO for viral replication assay.
