# Supplementary material for: A proteome-scale map of the SARS-CoV-2–human contactome
Source: Nat Biotechnol. 2022 Oct 10;41(1):140–9. doi: 10.1038/s41587-022-01475-z (PMC9849141; doi:10.1038/s41587-022-01475-z)
Supplement: Supplementary file 2 — Reporting Summary [file 41587_2022_1475_MOESM2_ESM.pdf]

nature portfolio

Corresponding author(s): Caroline Demeret, Marc Vidal, Michael A. Calderwood, Frederick P. Roth and Pascal Falter-Braun

Last updated by author(s): Aug 8, 2022

Reporting Summary

Nature Portfolio wishes to improve the reproducibility of the work that we publish. This form provides structure for consistency and transparency in reporting. For further information on Nature Portfolio policies, see our Editorial Policies and the Editorial Policy Checklist.

Statistics

For all statistical analyses, confirm that the following items are present in the figure legend, table legend, main text, or Methods section.

n/a	Confirmed	
☐	☒	The exact sample size (*n*) for each experimental group/condition, given as a discrete number and unit of measurement
☐	☒	A statement on whether measurements were taken from distinct samples or whether the same sample was measured repeatedly
☐	☒	The statistical test(s) used AND whether they are one- or two-sided<br>*Only common tests should be described solely by name; describe more complex techniques in the Methods section.*
☐	☒	A description of all covariates tested
☐	☒	A description of any assumptions or corrections, such as tests of normality and adjustment for multiple comparisons
☐	☒	A full description of the statistical parameters including central tendency (e.g. means) or other basic estimates (e.g. regression coefficient) AND variation (e.g. standard deviation) or associated estimates of uncertainty (e.g. confidence intervals)
☐	☒	For null hypothesis testing, the test statistic (e.g. *F*, *t*, *r*) with confidence intervals, effect sizes, degrees of freedom and *P* value noted<br>*Give P values as exact values whenever suitable.*
☒	☐	For Bayesian analysis, information on the choice of priors and Markov chain Monte Carlo settings
☒	☐	For hierarchical and complex designs, identification of the appropriate level for tests and full reporting of outcomes
☒	☐	Estimates of effect sizes (e.g. Cohen's *d*, Pearson's *r*), indicating how they were calculated

Our web collection on statistics for biologists contains articles on many of the points above.

Software and code

Policy information about availability of computer code

Data collection	Bowtie2 2.2.3<br>Illumina bcl2fastq 2.20.0.422<br>Python 2.7.10<br>Custom code for Y2H screening pipeline<br>Tissue Atlas dataset from the Human Protein Atlas database (version: 2021.04.09)
Data analysis	Cytoscape 3.8.2<br>Matlab 2016a<br>gProfiler (Database versions: Ensembl 104, Ensembl Genomes 51 and Wormbase ParaSite 15)<br>R 3.6.1<br>linkcomm R package<br>Metascape web-platform<br>Pfam release 34.0<br>FlowJo Software 10<br>MAGMA v1.09a<br>MikroWin 2010<br>Graphpad Prism v. 7.04,<br>Graphpad Prism v. 9,<br>AmiGO 2

For manuscripts utilizing custom algorithms or software that are central to the research but not yet described in published literature, software must be made available to editors and reviewers. We strongly encourage code deposition in a community repository (e.g. GitHub). See the Nature Portfolio guidelines for submitting code & software for further information.

Data

Policy information about availability of data

All manuscripts must include a data availability statement. This statement should provide the following information, where applicable:

- Accession codes, unique identifiers, or web links for publicly available datasets
- A description of any restrictions on data availability
- For clinical datasets or third party data, please ensure that the statement adheres to our policy

The PPI data from this publication have been submitted to the IMEx (http://www.imexconsortium.org) consortium through IntAct and assigned the identifier IM-28880104. All data from the study are included in the manuscript and associated files. Source data are provided with this paper.
The following data were obtained from the respective original publications: phosphorylation changes upon SARS-CoV-2 infection10,11; RNA-binding changes upon SARS-CoV-2 infection12; AP-MS virus-host association data: Gordon et al.14,15, Stukalov et al.10, Li et al.13, Nabeel-Shah et al.16; BioID virus-host proximity data: Laurent et al.17, St-Germain et al.19 Samavarchi-Tehrani et al.18; Human expression data: Human Proteome Atlas22, SARS-CoV-2 organotropism23,99; human host interactome: HuRI27; GWAS data for severe COVID-19 illness33,34;
GWAS summary statistics for 114 traits: doi:10.5281/ZENODO.3518299; Interaction data for other viruses were downloaded from IntAct9 (version: April 28, 2020).
Publication counts downloaded from gene2pubmed (NCBI), on 2021-11-16.

Field-specific reporting

Please select the one below that is the best fit for your research. If you are not sure, read the appropriate sections before making your selection.

☒ Life sciences ☐ Behavioural & social sciences ☐ Ecological, evolutionary & environmental sciences

For a reference copy of the document with all sections, see nature.com/documents/nr-reporting-summary-flat.pdf

Life sciences study design

All studies must disclose on these points even when the disclosure is negative.

Sample size	Sample size for PPI validation assay experiments was chosen based on a 95% CI value of 5% inputting and an expected recovery rate of 20%, from previous similar experiments, using the standard error of proportion. For IntraSCI the full dataset was tested. For all cell biological assays 3 - 8 biological repeats were done. Many years of experience have convinced us that this produces robust results.
Data exclusions	No data were excluded.
Replication	All attempts at replication were successful. For Y2H, 3 fold-replication is part of the standard quality control pipeline. In all cell biology experimnets, i.e. viral replication assays and NF-kB reporter activation assays, biological results were replicated at least twice (3 biological repeats).
Randomization	Randomization for network permutations was done while maintaining network topology (maintaining the number of interactions but shuffling the edges). yN2H validations were done with a fully randomized setup to avoid experimental bias and eliminate covariates, i.e. all pairs, except hsPRSv2/hs/RRv2, were randomly distributed across the plates. For experimental reasons, and because extensive prior data are available for hsPRSv2/hs/RRv2 benchmark pairs these were randomly mixed among each other, but separate from other benchmark and test pairs. For all cell culture and validation experiments, all samples were treated by the same researcher in parallel in random order to eliminate covariates. In all experiments, negative controls from the same experimental batch were used as initial reference.
Blinding	Y2H screens were done in a fully random and unbiased manner. Manual scoring of Y2H pairwise tests was blinded. Experimental design of yN2H validation was blinded during the experiment. For cell culture experiments with small sample sizes blinding was not possible, but all experiments are internally controlled to reduce researcher biased experimental differences.

Reporting for specific materials, systems and methods

We require information from authors about some types of materials, experimental systems and methods used in many studies. Here, indicate whether each material, system or method listed is relevant to your study. If you are not sure if a list item applies to your research, read the appropriate section before selecting a response.

Materials & experimental systems

n/a	Involved in the study
☐	☒ Antibodies
☐	☒ Eukaryotic cell lines
☒	☐ Palaeontology and archaeology
☒	☐ Animals and other organisms
☒	☐ Human research participants
☒	☐ Clinical data
☒	☐ Dual use research of concern

Methods

n/a	Involved in the study
☒	☐ ChIP-seq
☒	☐ Flow cytometry
☒	☐ MRI-based neuroimaging

Antibodies

Antibodies used

1. Anti-b-Actin (SCBT, sc-47778, RRID: AB_2714189)
2. anti-FLAG M2 (Sigma Aldrich, F3165, RRID:AB_259529)
3. anti-HA (Sigma-Aldrich, 11583816001, RRID:AB_514505)
4. Secondary antibody (JacksonImmuno Research, Jim-715-035-150)

Validation

The antibodies have been validated by the manufacturers and were not revalidated in this study.

Eukaryotic cell lines

Policy information about cell lines

Cell line source(s)

1. A549 (purchased from ATCC)
2. Hek293 (RRID: CVCL_0045, purchased from DSMZ)
3. Vero E6 (ATCC CRL-1586), kindly provided by Prof. Nicolas Gillet (University of Namur, Belgium)
4. Yeast strains Y8800 and Y8930: the same strains used in the many previous studies (Rolland, et al. Cell. 2014 Nov 20;159(5):1212-1226.)
5. Yeast strains - RY3011 and RY3031: Derived from Y8800 and Y8930 in Dr. Fritz Roth's lab at the University of Toronto

Authentication

Vero E6 cells were re-authenticated by RNA-Seq. Other cell lines were purchased from either ATCC or DSMZ and not re-authenticated.

Mycoplasma contamination

All cell lines were tested by PCR for mycoplasma contamination and found to be negative

Commonly misidentified lines
(See ICLAC register)

No commonly misidentified cell lines were used.
