# Supplementary figures and images for: A proteome-scale map of the SARS-CoV-2–human contactome
Source: Nat Biotechnol. 2022 Oct 10;41(1):140–9. doi: 10.1038/s41587-022-01475-z (PMC9849141; doi:10.1038/s41587-022-01475-z)

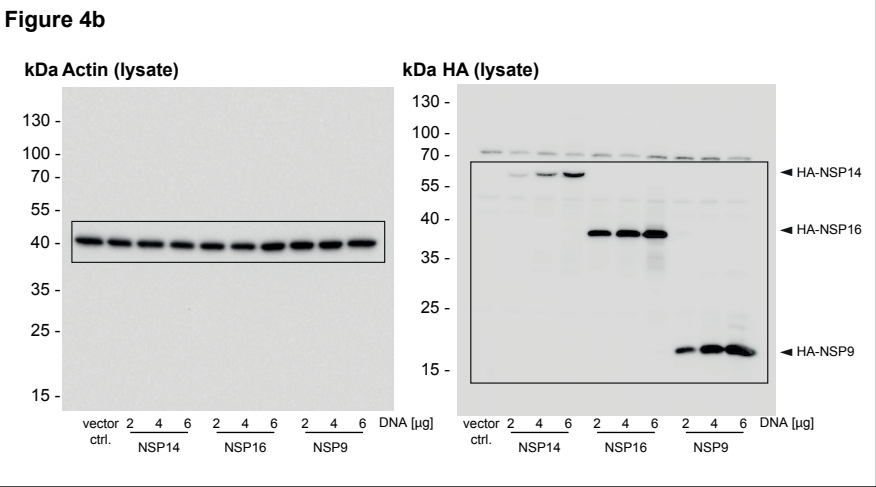

Supplement: Source Data Fig. 4 — c: unprocessed western blot. [file 41587_2022_1475_MOESM18_ESM.pdf]

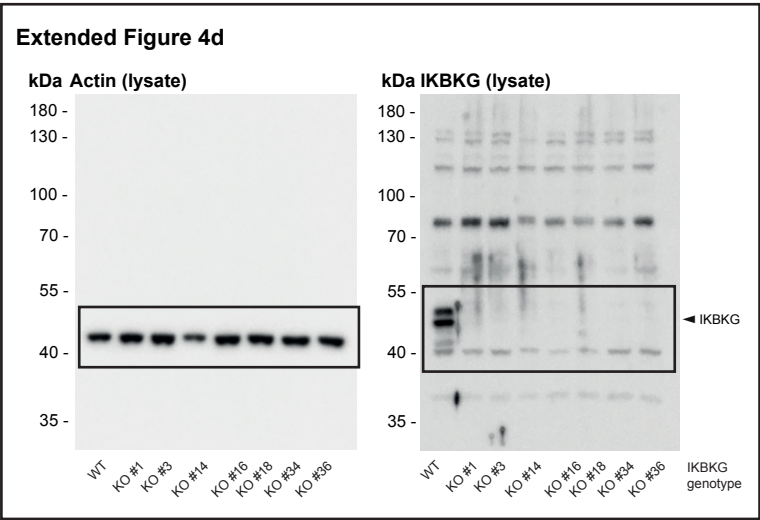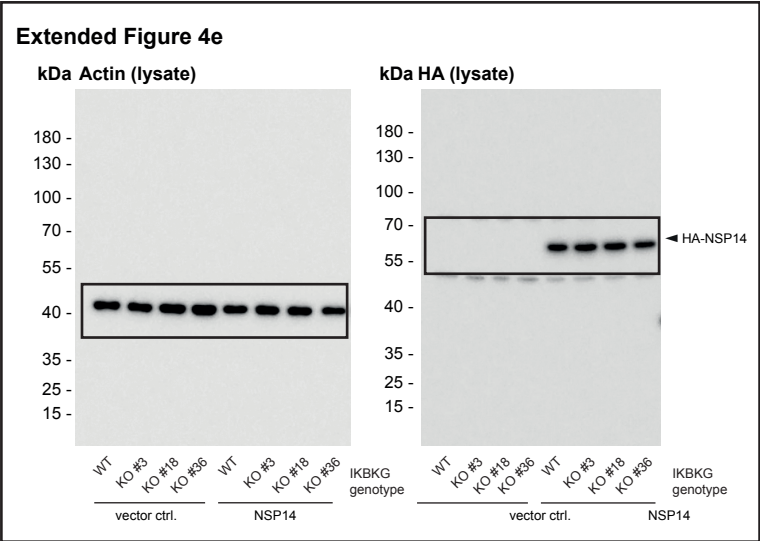

Supplement: Source Data Extended Data Fig. 4 — d,e: unprocessed western blot. [file 41587_2022_1475_MOESM19_ESM.pdf]
